# On the genetic basis of tail-loss evolution in humans and apes

**DOI:** 10.1038/s41586-024-07095-8

**Published:** 2024-02-28

**Authors:** Bo Xia, Weimin Zhang, Guisheng Zhao, Xinru Zhang, Jiangshan Bai, Ran Brosh, Aleksandra Wudzinska, Emily Huang, Hannah Ashe, Gwen Ellis, Maayan Pour, Yu Zhao, Camila Coelho, Yinan Zhu, Alexander Miller, Jeremy S. Dasen, Matthew T. Maurano, Sang Y. Kim, Jef D. Boeke, Itai Yanai

**Affiliations:** 1 Institute for Computational Medicine, NYU Langone Health, New York, NY USA; 2 Institute for Systems Genetics, NYU Langone Health, New York, NY USA; 3 Gene Regulation Observatory, Broad Institute of MIT and Harvard, Cambridge, MA USA; 4 Society of Fellows, Harvard University, Cambridge, MA USA; 5 Department of Biology, Pennsylvania State University, University Park, PA USA; 6 Department of Neuroscience and Physiology, NYU Langone Health, New York, NY USA; 7 Department of Pathology, NYU Langone Health, New York, NY USA; 8 Department of Biochemistry and Molecular Pharmacology, NYU Langone Health, New York, NY USA; 9 Department of Biomedical Engineering, NYU Tandon School of Engineering, Brooklyn, NY USA

**Keywords:** Mobile elements, Body patterning, Evolutionary developmental biology, Comparative genomics, Evolutionary genetics

## Abstract

The loss of the tail is among the most notable anatomical changes to have occurred along the evolutionary lineage leading to humans and to the ‘anthropomorphous apes’^1–3^, with a proposed role in contributing to human bipedalism^4–6^. Yet, the genetic mechanism that facilitated tail-loss evolution in hominoids remains unknown. Here we present evidence that an individual insertion of an Alu element in the genome of the hominoid ancestor may have contributed to tail-loss evolution. We demonstrate that this Alu element—inserted into an intron of the *TBXT* gene^7–9^—pairs with a neighbouring ancestral Alu element encoded in the reverse genomic orientation and leads to a hominoid-specific alternative splicing event. To study the effect of this splicing event, we generated multiple mouse models that express both full-length and exon-skipped isoforms of *Tbxt*, mimicking the expression pattern of its hominoid orthologue *TBXT*. Mice expressing both *Tbxt* isoforms exhibit a complete absence of the tail or a shortened tail depending on the relative abundance of *Tbxt* isoforms expressed at the embryonic tail bud. These results support the notion that the exon-skipped transcript is sufficient to induce a tail-loss phenotype. Moreover, mice expressing the exon-skipped *Tbxt* isoform develop neural tube defects, a condition that affects approximately 1 in 1,000 neonates in humans^10^. Thus, tail-loss evolution may have been associated with an adaptive cost of the potential for neural tube defects, which continue to affect human health today.

## Abstract (web summary)

An insertion of an Alu element into an intron of the *TBXT* gene is identified as a genetic mechanism of tail-loss evolution in humans and apes, with implications for human health today.

## Main

**Fig. 1:**
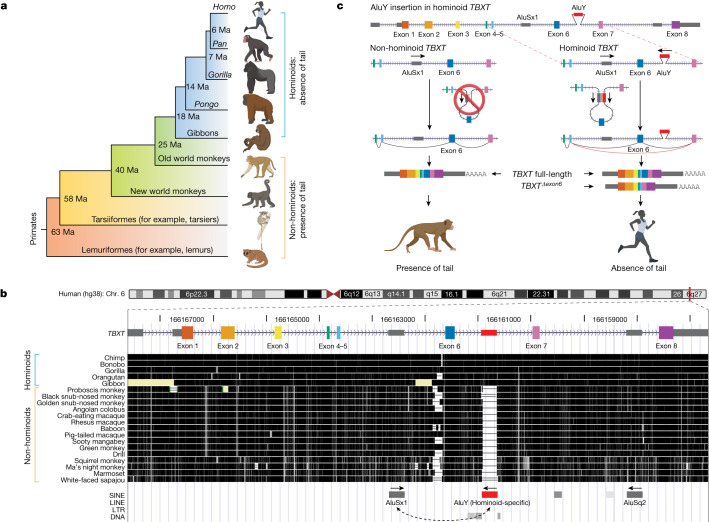
Evolution of tail loss in hominoids. **a**, Tail phenotypes across the primate phylogenetic tree. Ma, millions of years ago. **b**, UCSC Genome browser view^51^ of the conservation score through multi-species alignment at the *TBXT* locus across primate genomes. Exon numbering of human *TBXT* follows a conventional order across species without including the 5′ untranslated region exon. The hominoid-specific AluY element is highlighted in red. LINE, long interspersed nuclear element; LTR, long terminal repeat; SINE, short interspersed nuclear element. **c**, Schematic of the proposed mechanism of tail-loss evolution in hominoids. Primate images in **a** and **c** were created using BioRender (https://biorender.com).

The tail appendage varies widely in its morphology and function across vertebrate species^4,6^. For primates in particular, the tail is adapted to a range of environments, with implications for the style of locomotion of the animal^11,12^. The New World howler monkeys, for example, evolved a prehensile tail that helps with the grasping or holding of objects while occupying arboreal habitats^13^. Hominoids—which include humans and the apes—however, lost their external tail during evolution. The loss of the tail is inferred to have occurred around 25 million years ago when the hominoid lineage diverged from the ancient Old World monkeys (Fig. 1a), leaving only 3–5 caudal vertebrae to form the coccyx, or tailbone, in modern humans^14^.

It has long been speculated that tail loss in hominoids contributed to orthograde and bipedal locomotion, the evolutionary occurrence of which coincided with the loss of the tail^15–17^. Yet, the genetic mechanism that facilitated either tail-loss evolution or orthograde and bipedal locomotion in hominoids remains unknown. Recent progress in primate genome sequencing projects have made it possible to infer causal links between genotypic and phenotypic changes^18–20^, and have enabled the search for hominoid-specific genetic elements that control tail development^21^. Moreover, developmental genetics studies of vertebrates have led to the elucidation of the gene regulatory networks that underlie tail development^21,22^. For example, the Mouse Genome Informatics (MGI) database includes more than 100 genes identified from natural mutants and induced mutagenesis studies relating to the absence or shortening of the tail phenotype^22,23^ (Supplementary Data 1 and Methods). Expression of these genes, including the core factors for inducing mesoderm and definitive endoderm such as *Tbxt* (also called *T* or *Brachyury*), *Wnt3a* and *Msgn1*, is enriched in the development of the primitive streak and posterior body formation. Although perturbations of these genes may lead to the shortening or complete absence of the tail, the causal genetic changes that drove the evolution of tail-loss in hominoids remains unknown. Understanding the genetics of tail loss in hominoids may provide insight into the evolutionary pressure that led to human traits such as bipedalism.

## A hominoid-specific intronic AluY in *TBXT*

**Extended Data Fig. 1:**
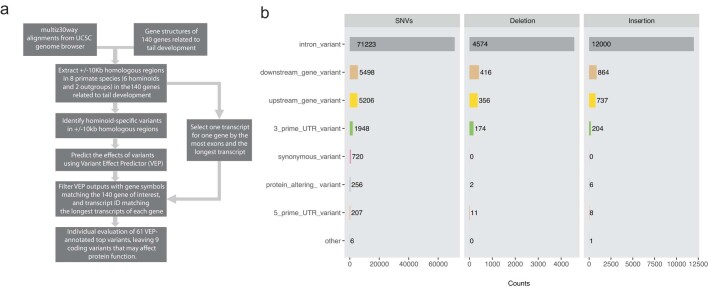
Comparative genomics analyses of hominoid-specific variants in the genes related to tail development. **a**, Workflow of the comparative genomics analyses (Methods). **b**, Summary of all detected hominoid-specific variants with respect to the outgroup species.

With the goal of identifying genetic variants associated with the loss of the tail in hominoids, we initially screened 31 human genes—and their primate orthologues—for which mutations are associated with the absence of an external tail (MGI database annotation ‘absent tail’; Supplementary Data 1 and Methods). We first examined protein sequence conservation between the hominoid genomes and their closest sister lineage, the Old World monkeys (Cercopithecidae). Failing to detect candidate variants in the coding regions of this gene set, we expanded the search in two ways: (1) adding 109 genes for which mutation in their mouse orthologues includes tail-reduction phenotypes annotated in the MGI as ‘vestigial tail’ or ‘short tail’; and (2) systematically screening for hominoid-specific variants in the entire gene region and their 10 kb upstream and downstream sequences (Supplementary Data 1 and Methods). Together, we detected 85,064 single nucleotide variants (SNVs), 5,533 deletions and 13,820 insertions that are hominoid-specific (Extended Data Fig. 1 and Supplementary Data 1–4). Among these changes, we identified nine protein-sequence altering variants—seven missense variants and two in-frame deletions—with predicted impacts on function (Supplementary Data 1 and Methods). However, these variants originated from genes that after perturbation influence more general growth and developmental defects as opposed to specifically tail-reduction phenotypes (Supplementary Data 1). Although we were not able to exclude the possibility that these variants might have contributed to tail-loss evolution in hominoids, we did not find additional supporting evidence to prioritize their experimental validation as a plausible genetic mechanism.

Examining non-coding hominoid-specific variants among the genes related to tail development (Methods), we recognized an Alu element in the sixth intron of the hominoid *TBXT* gene^7,8^ (Fig. 1b). This element had the following notable combination of features: (1) a hominoid-specific phylogenetic distribution; (2) presence in a gene known for its involvement in tail formation; and (3) proximity and orientation relative to a neighbouring Alu element. First, this particular hominoid-specific Alu element is from the AluY subfamily, a relatively ‘young’ but not human-specific subfamily shared among the genomes of hominoids and Old World monkeys. Moreover, the inferred insertion time—given the phylogenetic distribution (Fig. 1a)—coincides with the evolutionary period when early hominoids lost their tails^24^. Second, *TBXT* encodes a highly conserved transcription factor crucial for mesoderm and definitive endoderm formation during embryonic development^9,25–27^. Heterozygous mutations in the coding regions of *TBXT* orthologues in tailed animals such as mouse^7,28^, Manx cat^29^, dog^30^ and zebrafish^31^ lead to the absence or reduced forms of the tail, and homozygous mutants are typically non-viable.

**Extended Data Fig. 2:**
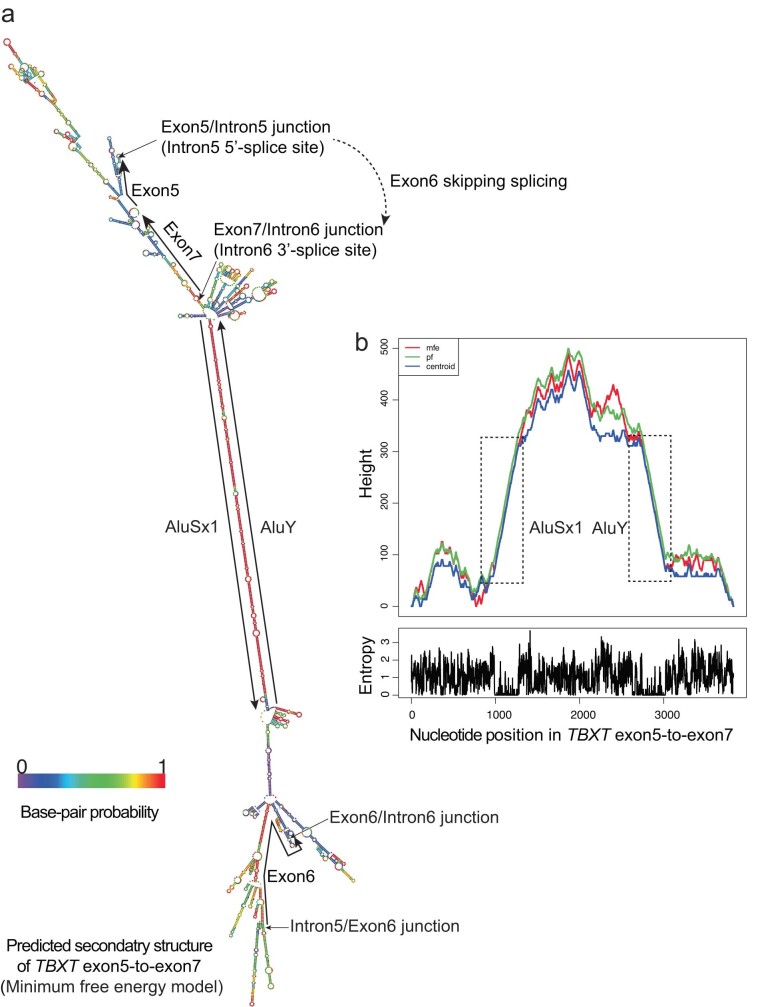
RNA structure prediction. **a**, Predicted RNA secondary structure of the *TBXT* exon5-to-exon7 sequence using the RNAfold algorithm of the ViennaRNA package^35^. The paired AluY-AluSx1 region was highlighted. **b**, Mountain plot of the RNA secondary structure prediction, showing the ‘height’ in predicted secondary structure across the nucleotide positions. Height was computed as the number of base pairs enclosing the base at a given position. Overall, the AluSx1 and AluY regions were predicted to form helices with high probability (low entropy).

**Extended Data Fig. 3:**
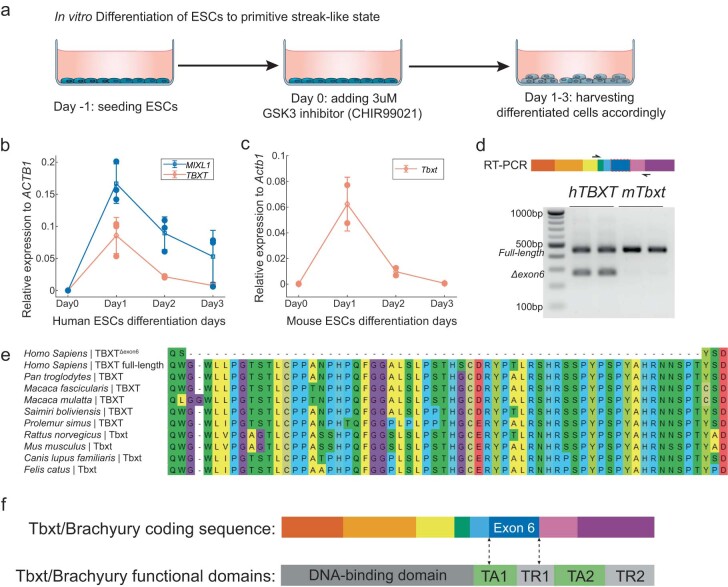
Analyses of *TBXT* isoforms. **a**, In vitro differentiation of human and mouse ESCs for inducing *TBXT*/*Tbxt* expression. Human and mouse ESCs differentiation assay was adapted from Xi et al. (2017)^36^ and Pour et al. (2019)^37^, respectively. Schematic adapted from icons created by Marcel Tisch via bioicons.com. **b**, Quantitative RT-PCR (RT-qPCR) of *TBXT* and *MIXL1* expression during hESC differentiation, indicating correct induction of mesodermal gene expression program^36^. **c**, Quantitative RT-PCR of *Tbxt* expression during mESC differentiation. Data in **b** and **c** were presented as mean +/− standard deviation of the relative gene expression levels. Sample number n = 3 represents RT-qPCR results from three biologically independent RNA samples, with each data point averaged from 3 technical replicates in quantitative PCR. **d**, RT-PCR of *TBXT*/*Tbxt* transcripts in human and mouse differentiated ESCs, highlighting the *Δexon6* splicing isoform unique to human. RT-PCR results were presented as biological duplicates. **e**, Protein sequence alignment of TBXT*-*exon6 region in the representative mammals. All presented animals have tails except human and chimpanzee. **f**, The exon 6-coded peptide of Tbxt protein overlaps with large fractions of two transcription regulation domains. TA, transcription activation; TR, transcription repression. Functional domain annotation of mouse Tbxt protein was adapted from Kispert et al. (1995)^9^.

Third, we inferred that the AluY insertion may mediate an alternative splicing (AS) event of the hominoid *TBXT* in an unusual way. This AluY element is not inserted in the vicinity of a splice site; instead, it is >500 bp from exon 6 of *TBXT*, the nearest coding exon (Fig. 1b). As such, it would not be expected, by itself, to lead to an AS event, as found for other individual intronic Alu elements near exon boundaries that directly affect splicing^32–34^. However, we noted the presence of another Alu element (AluSx1) in the reverse orientation in intron 5 of *TBXT* that is shared among all monkeys and apes (simians). Together, the AluY and AluSx1 elements form an exon-flanking inverted repeat pair (Fig. 1b). We therefore posited that during transcription, the hominoid-specific AluY element pairs with the simian-shared AluSx1 element to form a stem–loop structure in *TBXT* pre-mRNA and traps exon 6 in the loop (Fig. 1c). An inferred model of the RNA secondary structure supported the interaction between these two Alu elements^35^ (Extended Data Fig. 2). The secondary structure of the transcript may conjoin the splice donor and receptor site of exons 5 and 7, respectively, and promote the skipping of exon 6, thereby leading to a hominoid-specific and in-frame AS isoform: *TBXT*^*Δexon6*^ (Fig. 1c). We validated the existence of the *TBXT*^*Δexon6*^ transcript in humans and its corresponding absence in mice, which lacks both Alu elements, using a system for embryonic stem (ES) cell in vitro differentiation that induces *TBXT* expression similar to that present in the primitive streak of the embryo^36,37^ (Extended Data Fig. 3a–d and Supplementary Table 1). Considering the high conservation of *TBXT* exon 6 and its potential transcriptional regulation function but not the DNA-binding function^9,38^ (Extended Data Fig. 3e,f), we proposed that the AluY-insertion-induced TBXT(Δexon6) isoform protein disrupts tail elongation during embryonic development, which then contributes to the reduction or loss of an external tail (Fig. 1c).

## AluY insertion in *TBXT* induces AS

**Fig. 2:**
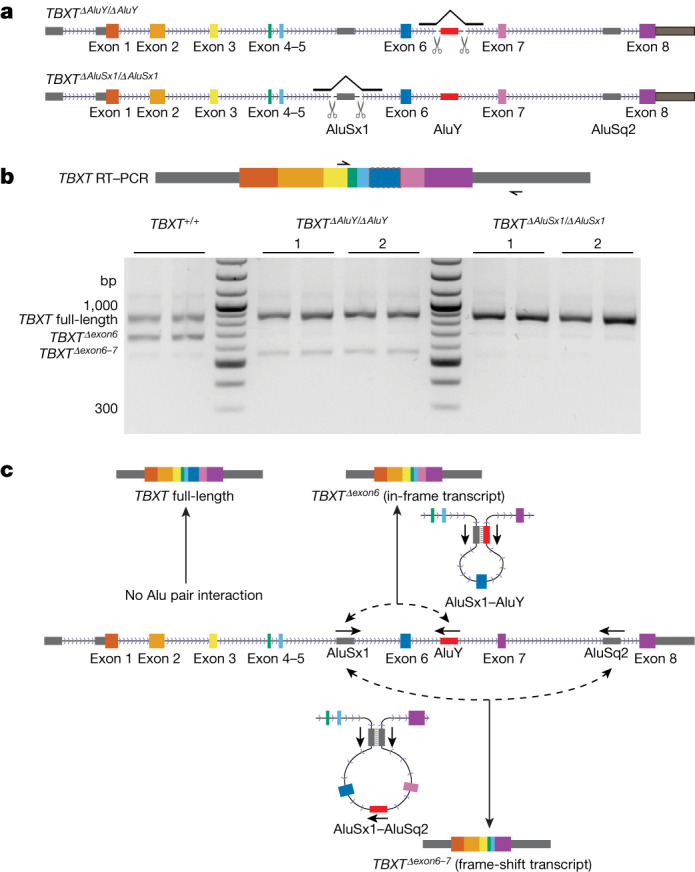
Both AluY and AluSx1 are required for inducing alternative splicing of *TBXT*. **a**, CRISPR-generated homozygous knockouts of the AluY element in *TBXT* intron 6 (top, *TBXT*^*ΔAluY/ΔAluY*^) and AluSx1 element in intron 5 (bottom, *TBXT*^*ΔAluSx1/ΔAluSx1*^) in human ES cells. **b**, RT–PCR results of *TBXT* transcripts isolated from differentiated human ES cell of wild-type, *TBXT*^*ΔAluY/ΔAluY*^ and *TBXT*^*ΔAluSx1/ΔAluSx1*^ genotypes. Each mutant line included two independent clones. All RT–PCR results were performed in technical duplicates. **c**, A schematic of inferred Alu interactions and the corresponding *TBXT* transcripts, which indicate that an AluY–AluSx1 interaction leads to the *TBXT*^*Δexon6*^ transcript. The *TBXT*^*Δexon6–7*^ transcript may stem from an AluSx1–AluSq2 interaction.

**Extended Data Fig. 4:**
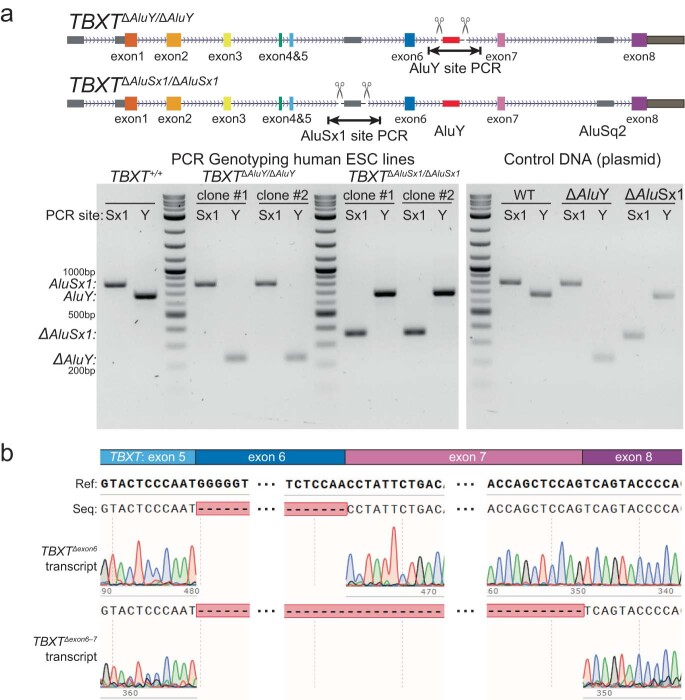
Validation of *Alu*-deletion hESC clones and the expressed *TBXT* isoforms. **a**, PCR validation of the hESC clones with deletions of AluY or AluSx1 in *TBXT*. PCR validation for each sample were performed in pairs, each amplifying both AluSx1 locus (Sx1) and the AluY locus (Y) using primers that bind the two flanking sequences of the targeted region, respectively. Each genotype included two independent clones of AluY deletion or AluSx1 deletion, corresponding to the two clones presented in Fig. 2b. **b**, Sanger sequencing of the *TBXT*^*Δ*^^*exon6*^ and *TBXT*^*Δ*^^*exon6-7*^ transcripts detected in Fig. 2b. The sequencing results were aligned to the *TBXT* full length mRNA sequence.

To test whether AluY—and its interacting counterpart AluSx1—are both required to induce the hominoid-specific AS of *TBXT*, we used CRISPR–Cas9 tool to generate human ES cell lines that individually deleted the hominoid-specific AluY element or the AluSx1 element (Fig. 2a, Extended Data Fig. 4a and Supplementary Tables 2–4). We adapted the human ES cell in vitro differentiation system to mimic the expression of *TBXT* in the embryo^36^ (Extended Data Fig. 3a). Deleting AluY almost completely eliminated the generation of the *TBXT*^*Δexon6*^ isoform transcript (Fig. 2b, middle). Similarly, deleting the interacting partner AluSx1 was sufficient to repress this alternatively spliced isoform (Fig. 2b, right). These results support the notion that the hominoid-specific AluY insertion induces a new *TBXT*^*Δexon6*^ AS isoform through an interaction with the neighbouring simian-shared AluSx1 element (Fig. 2c, top).

Notably, wild-type differentiated human ES cells also expressed a minor, previously un-annotated transcript that excludes both exons 6 and 7, which led to a frameshift and early truncation at the protein level (Fig. 2b, left, and Extended Data Fig. 4b). Whereas deleting AluY slightly enhanced the abundance of this *TBXT*^*Δexon6–7*^ transcript, deleting AluSx1 in intron 5 eliminated this transcript (Fig. 2b). This result may be best explained by a secondary interaction of the AluSx1 element with a distal and inverted AluSq2 element in intron 7. In this scenario, the secondary interaction would occur at a lower probability than the AluY–AluSx1 interaction pair (Fig. 2c, bottom). It is noteworthy that the distance between the *Alu*Sx1–AluY pair is substantially shorter (1,448 bp) than the AluSx1–AluSq2 distance (4,188 bp). Furthermore, the nascent transcript would favour formation of the former structure as there is a time period during which the AluSx1–AluY structure can form and the distal structure cannot; these factors could potentially explain the preferred formation of the *Δexon6* mRNA over *Δexon6–7* mRNA. These results provide further support to indicate that the interaction between intronic transposable elements induces AS of a key developmental transcriptional factor gene: *TBXT* (Fig. 2c).

## *Tbxt*^*Δexon6*^ expression induces tail loss

**Fig. 3:**
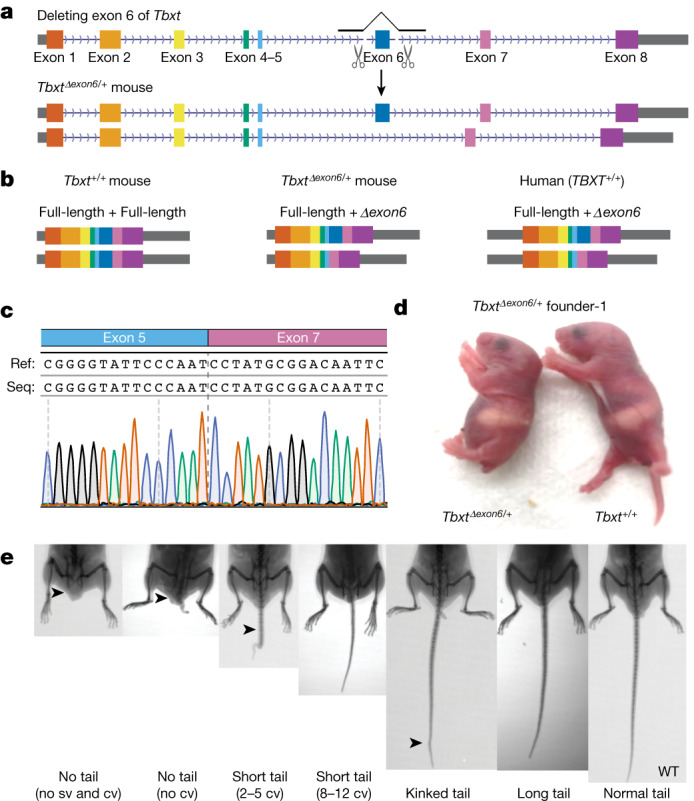
The *TBXT*^*Δexon6*^ isoform is sufficient to induce tail-loss phenotype. **a**, CRISPR design for generating the *Tbxt*^*Δexon6/+*^ heterozygous mouse model. **b,** Schematic of *TBXT* transcripts in human and mouse models. *Tbxt*^*Δexon6/+*^ mouse mimics *TBXT* gene expression products in humans. **c**, Sanger sequencing of the RT–PCR product confirmed that deleting exon 6 in mouse *Tbxt* leads to correct splicing by fusing exons 5 and 7. **d**, A representative *Tbxt*^*Δexon6/+*^ founder mouse (day 1) exhibiting a no-tail phenotype. Two additional founder mice are shown in Extended Data Fig. 5. **e**, *Tbxt*^*Δexon6/+*^ mice exhibit heterogeneous tail phenotypes, varying from no tail to long tails. cv, caudal vertebrae; sv, sacral vertebrae; WT, wild type; arrowheads highlight differences in tail phenotypes.

**Extended Data Fig. 5:**
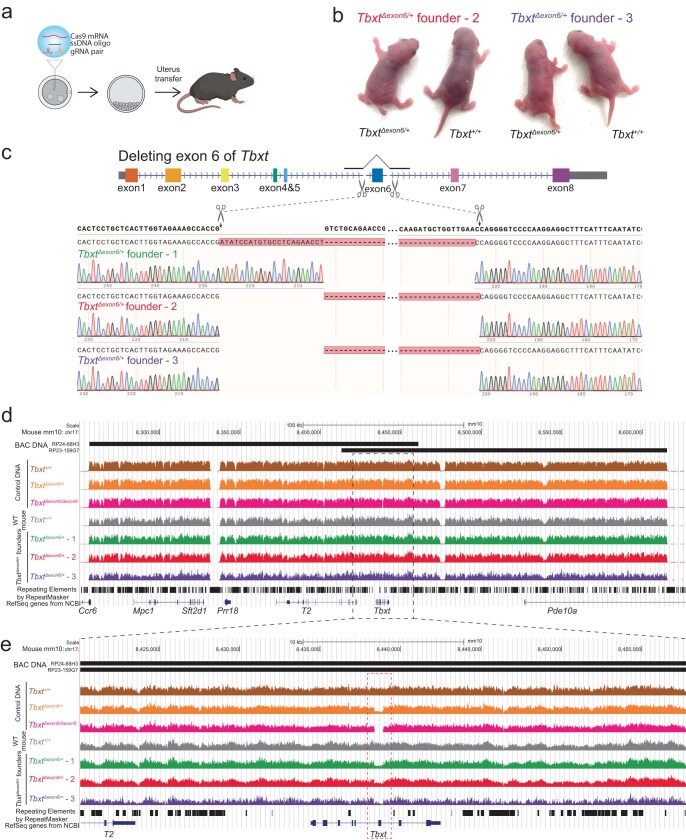
*Tbxt*^*Δexon6/+*^ founder mice generated through zygotic CRISPR/Cas9 targeting approach. **a**, Schematic of zygotic injection of CRISPR/Cas9 reactions. **b**, Two more *Tbxt*^*Δexon6/+*^ founder mice (in addition to the one shown in Fig. 3d) indicating an absence or reduced form of the tail. **c**, Sanger sequencing of the exon 6-deleted allele isolated from the genomic DNA of *Tbxt*^*Δexon6/+*^ founder mice. Founder 1 had an unexpected insertion of 23 base pairs at the CRISPR cutting site in the original intron 5 of *Tbxt*. Both founder 2 and 3 had the exact fusion between the two CRISPR cutting sites in introns 5 and 6. **d**–**e**, Capture-seq analyses at the *Tbxt* locus of founder mice did not detect off-target mutations. A zoomed-in view of the Capture-seq results at the *Tbxt* locus highlights the CRISPR-mediated exon 6 deletion (**e**). Capture-seq baits were generated from bacterial artificial chromosomes (RP24-88H3 and RP23-159G7). The shallow-covered regions are typically repeat sequences in the mouse genome and are consistent across samples. Control DNAs were obtained from wild-type or *Tbxt*^*Δexon6/Δexon6*^ mESCs, and the heterozygous sample came from a 1:1 mixture of genomic DNA from wild-type and *Tbxt*^*Δexon6/Δexon6*^ mESCs.

To test whether the *TBXT*^*Δexon6*^ isoform is sufficient to induce tail loss, we first used zygotic CRISPR targeting to generate a heterozygous mouse model (*Tbxt*^*Δexon6/+*^) that simultaneously expresses the *Tbxt*^*Δexon6*^ transcript and its full-length transcript (Fig. 3a,b, Extended Data Fig. 5a and Methods). *TBXT* is highly conserved in vertebrates, and human and mouse protein sequences share 91% identity with a similar exon and intron architecture^8^. We reasoned that we could simulate a *Δexon6* isoform by deleting exon 6 in mouse *Tbxt* and force the splicing of exon 5 to exon 7 (Fig. 3b,c). The *Tbxt*^*Δexon6/+*^ mouse therefore provides a model of the expression of *TBXT* in humans, which expresses both full-length and Δexon6 isoforms (Figs. 2b and 3b,c).

The phenotypes of *Tbxt*^*Δexon6/+*^ mice exhibited strong but heterogeneous tail morphologies, including no-tail and short-tail phenotypes (Fig. 3d,e and Extended Data Fig. 5b,c). Specifically, 21 out of the 63 heterozygous mice showed tail phenotypes, whereas none of their 35 wild-type littermates showed phenotypes (Table 1). The incomplete penetrance of phenotypes among the heterozygotes was stable across generations and founder lines: no-tail or short-tailed (*Tbxt*^*Δexon6/+*^) parents gave birth to long-tailed *Tbxt*^*Δexon6/+*^ mice, whereas long-tailed (*Tbxt*^*Δexon6/+*^) parents gave birth to mice with varied tail phenotypes (Table 1 and Extended Data Fig. 5b,c). These results provide further evidence that the presence of *TBXT*^*Δexon6*^ is sufficient to induce tail loss.

**Table 1:** Genotype and phenotype analyses of the F_2_ mice generated from intercrossing *Tbxt*^*Δexon6/*+^ parents

Genotype	Total no. of F_2_ mice	No. of mice with tail phenotype	Tail phenotype			Intercross (type 1)^a^	Intercross (type 2)^a^
			No tail	Short tail	Kinked tail		
*Tbxt*^*∆exon6/∆exon6*^	0	0	0	0	0	0	0
*Tbxt*^*∆exon6/*+^	63	21	4	9	8	17 (7)^b^	46 (14)^b^
*Tbxt*^+/+^	35	0	0	0	0	7 (0)^b^	28 (0)^b^

Note that tail phenotypes were categorized into no tail, short tail, kinked tail and long tail, as exemplified in Fig. 3e.

^a^For type 1 intercrossing, at least one of the parent mice has no tail or is short-tailed. For type 2 intercrossing, both parent mice are long-tailed.

^b^Numbers in parentheses indicate the total number of mice with tail phenotypes.

To control for the possibility that zygotic CRISPR targeting induced off-targeting DNA changes at the *Tbxt* locus, we performed Capture-seq^39^ covering the *Tbxt* locus and about 200 kb of both upstream-flanking and downstream-flanking regions (Extended Data Fig. 5d,e). Capture-seq did not detect any off-target mutations at the *Tbxt* locus across three independent founder mice, which supports our conclusion that the observed tail phenotype in *Tbxt*^*Δexon6/+*^ mice was derived from the *Tbxt*^*Δexon6*^ genotype.

## Inserting intronic sequences in mouse *Tbxt*

Although the heterozygous mouse model (*Tbxt*^*Δexon6/+*^) showed that expression of both full-length and Δexon6 splice isoforms can produce a tail-loss phenotype, it does not assess whether AS is the mechanism for its generation. We therefore sought to test whether AS in human *TBXT* induced by the pairing of AluY and AluSx1 can be recapitulated in mouse *Tbxt*, and whether such a genetic change induces tail phenotypes.

**Extended Data Fig. 6:**
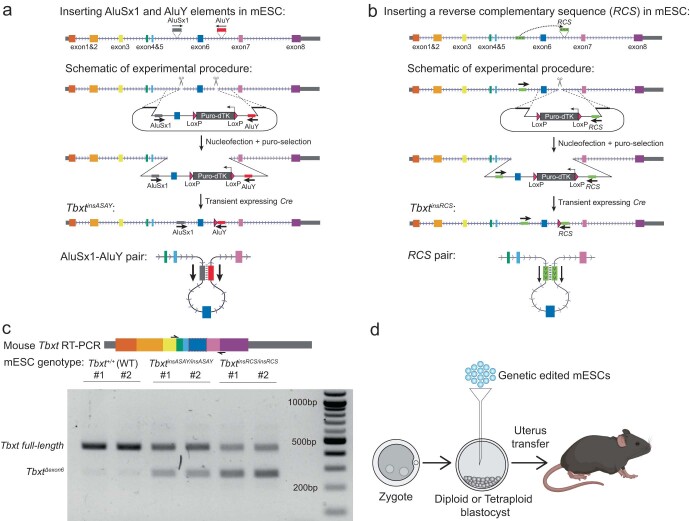
Engineering of inverted sequence pairs in mouse *Tbxt* induces alternative splicing. **a**–**b**, Schematics of mouse *Tbxt* gene structure with inserted human AluSx1-AluY pair (**a**, *Tbxt-insASAY*) or a designed intronic reverse complementary sequence of 297 bp (**b**, *Tbxt-insRCS*). The designed RCS insertion has the same length as AluY in human *TBXT*. In both designs, a two-step experimental procedure was adapted by first integrating the target elements with a selection cassette of puromycin-resistance and truncated thymidine kinase (*puro*-*ΔTK*) gene into the intron of mouse *Tbxt*, followed by removal of the selection cassette through transiently expressing *Cre* recombinase (Methods). **c**, *Tbxt* transcripts detected through RT-PCR using differentiated mouse ESC lines across wild-type (left), homozygous *Tbxt-insASAY* (*Tbxt*^*insASAY/insASAY*^, middle), and homozygous *Tbxt-insRCS* (*Tbxt*^*insRCS/insRCS*^, right) genotypes. RT-PCR results were presented as biological duplicates for each genotype. **d**, mESC injection into diploid or tetraploid blastocyst for generating *Tbxt*^*insASAY/insASAY*^ and *Tbxt*^*insRCS/insRCS*^ mouse models.

To that end, we first generated two mouse ES cell lines with *Tbxt* modifications, including simultaneously inserting the human AluY and AluSx1 elements into introns 6 and 5 of *Tbxt*, respectively, and inserting a reverse complementary sequence (RCS) from *Tbxt* intron 5 into intron 6 (Extended Data Fig. 6 and Supplementary Tables 2–4). For the first model, we simultaneously inserted the AluY and AluSx1 elements into mouse *Tbxt* (henceforth referred to as *Tbxt*^*insASAY*^) in an exon 6-flanking configuration that is analogous to the gene structure in human *TBXT* (Extended Data Fig. 6a). We designed a two-step strategy by first inserting two Alu elements together with a selection gene cassette of a puromycin-resistance gene and a truncated thymidine kinase gene (*puro-ΔTK*), flanked by *loxP* recombination motifs, for both positive selection and counter selection, respectively (Extended Data Fig. 6a). Following the identification of mouse ES cell clones with homozygous integration of the full construct, the selection gene cassette was removed by transiently expressing *Cre* recombinase in the selected clones through Δ*TK-*based counter selection (Extended Data Fig. 6a and Methods).

For the second mouse ES cell line, we adopted the same strategy but selected a 297 bp sequence endogenous to *Tbxt* intron 5—the same length as the human AluY—and then inserted its RCS into *Tbxt* intron 6, thus forming an inverted sequence pair like the AluSx1–AluY pair (referred as *Tbxt*^*insRCS*^; Extended Data Fig. 6b). We confirmed that both *Tbxt*^*insASAY/insASAY*^ and *Tbxt*^*insRCS/insRCS*^ ES cells expressed the *Tbxt*^*Δexon6*^ splicing isoform after differentiation (Extended Data Fig. 6c). Notably, the *Tbxt*^*insRCS/insRCS*^ ES cells expressed a higher percentage of *Tbxt*^*Δexon6*^ transcripts relative to the full-length transcript than that of *Tbxt*^*insASAY/insASAY*^ ES cells (Extended Data Fig. 6c). This result could be attributed to the sequence context difference and the higher sequence identity in the *Tbxt*^*insRCS*^ stem structure (297 out of 297 identical) than in the *Tbxt*^*insASAY*^ stem structure (228 out of 297). Together, these results demonstrate that the exon-skipping event caused by inverted Alu pairs flanking an exon do not require any specific Alu sequences, but can be caused by inverted sequence pairs of a completely different sequence.

## Abundance of *Tbxt* isoforms explains tail phenotypes

**Fig. 4:**
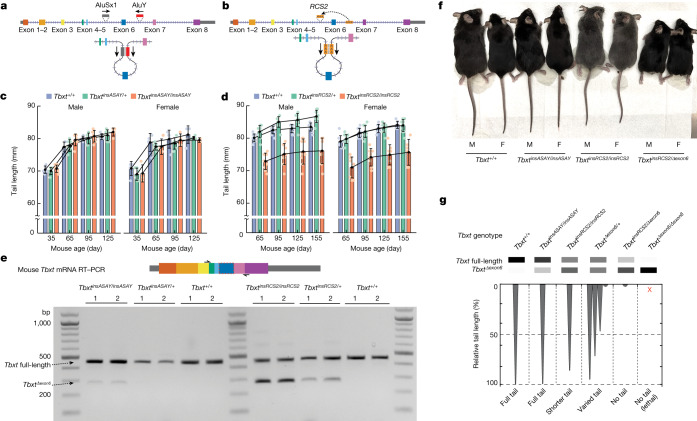
Introducing inverted intronic sequence pairs induces short-tail phenotypes in mouse models. **a**, Schematic of the mouse *Tbxt* gene structure with the inserted human AluSx1–AluY pair (*Tbxt*^*insASAY*^). The engineering of the *Tbxt*^*insASAY*^ model involved a two-step strategy specified in Extended Data Fig. 7a (Methods). **b**, Gene structure of the *Tbxt*^*insRCS2*^ model with an insertion of a 220 bp RCS from intron 6 to intron 5 of *Tbxt* (Methods). **c**,**d**, Tail length of *Tbxt*^*insASAY*^ mice (**c**) and *Tbxt*^*insRCS2*^ mice (**d**) across age, grouped by sex and genotypes. *Tbxt*^*+/+*^ is the wild type. Data in **c** and **d** are presented as the mean ± s.d. of tail length (mm) in the corresponding group. Each mouse group included 4–11 mice from multiple litters, with dots indicating individual data points of the group. **e**, Tailbud-expressed *Tbxt* transcripts detected by RT–PCR using E10.5 mouse embryos across genotypes from *Tbxt*^*insASAY*^ (left) or *Tbxt*^*insRCS2*^ (right) intercrossing experiments. RT–PCR results are presented as biological duplicates, with consistent results obtained from more independent embryos across genotypes. **f**, Representative tail phenotypes across mouse lines, including wild type, *Tbxt*^*insASAY/insASAY*^, *Tbxt*^*insRCS2/insRCS2*^ and *Tbxt*^*insRCS2/Δexon6*^. Each included both male (M) and female (F) mice. **g**, Summary schematic of the correspondence between the relative abundance of *Tbxt* isoforms in mice of different genotypes and their observed tail phenotypes.

**Extended Data Fig. 7:**
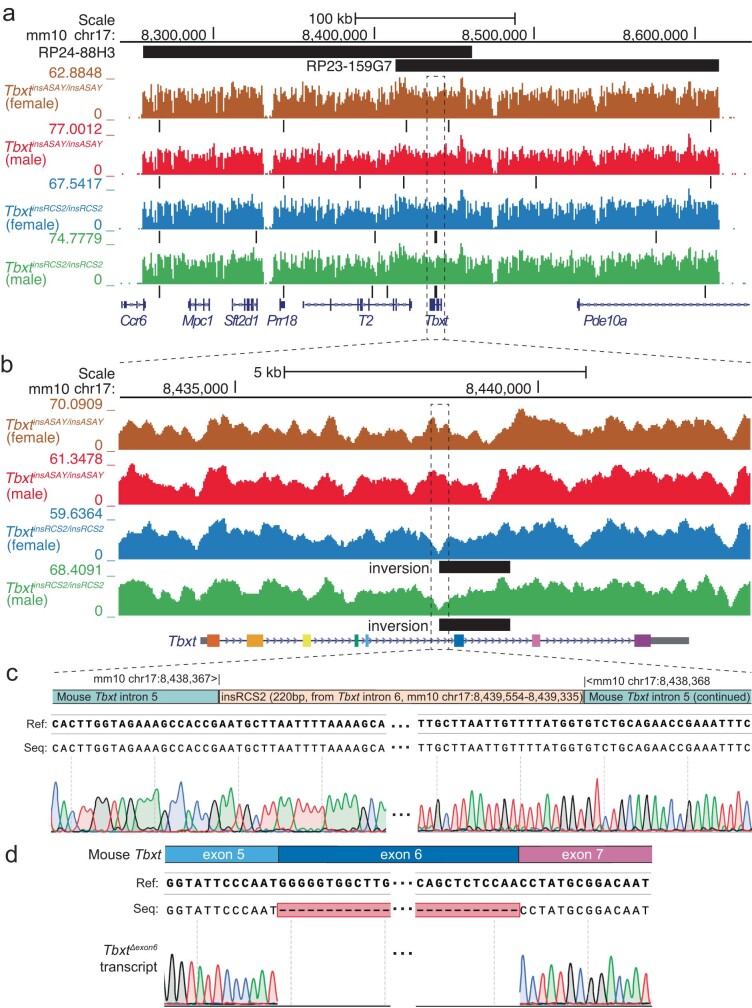
Validation of *Tbxt*^*insASAY/insASAY*^ and *Tbxt*^*insRCS2/insRCS2*^ homozygous mice. **a**-**b**, Capture-seq reads mapped to mouse reference genome mm10 at the full *Tbxt* locus (**a**) and a zoom-in view of *Tbxt* gene region (**b**) in *Tbxt*^*insASAY/insASAY*^ and *Tbxt*^*insRCS2/insRCS2*^ homozygous mice. Black ticks under each coverage track indicate the detected SNVs referring to the mm10 genome (**a**). The black bars in (**b**) indicate the detection of reads supporting an inversion. As expected, *Tbxt*^*insRCS2/insRCS2*^ samples incorporated an intronic sequence insertion, thus resembling an inversion event at the insertion site (**b**) due to forced mapping of the sequencing reads to reference genome. **c**, Sanger sequencing of *Tbxt*^*insRCS2/insRCS2*^ genomic sequence confirmed the inserted sequence and the exact insertion site. The inserted sequence constitutes a 220 bp sequence from *Tbxt-intron 6* (mm10 chr17: 8439335-8439554). **d**, Sanger sequencing of RT-PCR results using total RNA extracted from tailbud of *Tbxt*^*insRCS2/insRCS2*^ embryo at stage E10.5. The results correspond to Fig. 4e.

Next, we aimed to generate mouse models that incorporate the engineered *Tbxt*^*insASAY*^ and *Tbxt*^*insRCS*^ gene structures to study their tail phenotypes (Extended Data Fig. 6d and Methods). Through multiple experimental trials, we successfully generated one *Tbxt*^*insASAY*^ mouse line (Fig. 4a) but failed to derive any *Tbxt*^*insRCS*^ mouse lines. Instead, we serendipitously obtained another mouse line—henceforth called *Tbxt*^*insRCS2*^—that had an inserted 220 bp sequence from intron 6 into intron 5 of *Tbxt*, thereby resembling the *Tbxt*^*insRCS*^ design through forming a RCS pair flanking exon 6 (Fig. 4b, Extended Data Fig. 7a–c and Methods). Neither heterozygous nor homozygous *Tbxt*^*insASAY*^ mice showed obvious tail phenotypes in adulthood (Fig. 4c). However, homozygous *Tbxt*^*insRCS2*^ mice (*Tbxt*^*insRCS2/insRCS2*^) consistently had around 10% shorter tails relative to wild-type or heterozygous mice (Fig. 4d).

To gain insight into the distinct tail phenotypes in *Tbxt*^*insASAY*^ and *Tbxt*^*insRCS2*^ mice, we collected tailbud RNA samples from embryonic stage 10.5 (E10.5) embryos, when *Tbxt* is anticipated to influence tail development. Specifically, we processed RNA samples from litter-controlled wild-type, heterozygous and homozygous mice from intercrossed breeding pairs using heterozygous mice, followed by PCR with reverse transcription (RT–PCR) analyses of the expression patterns of *Tbxt* isoforms (Fig. 4e and Extended Data Fig. 7d). *Tbxt*^*insASAY*^ homozygous embryos expressed low levels of *Tbxt*^*Δexon6*^ transcript relative to the full-length transcript (Fig. 4e, left). By contrast, *Tbxt*^*insRCS2/insRCS2*^ embryos expressed higher levels of the *Tbxt*^*Δexon6*^ transcript than the *Tbxt* full-length transcript (Fig. 4e, right). As expected, in both lines, heterozygous embryos expressed lower levels of *Tbxt*^*Δexon6*^ transcript than their genotype-matched homozygous mice (Fig. 4e). These results suggest that the tail-length phenotype in *Tbxt*^*insASAY*^ and *Tbxt*^*insRCS2*^ mice can be explained by the relative abundance of *Tbxt*^*Δexon6*^ and *Tbxt* full-length transcripts.

It is important to note that the *Tbxt*^*insASAY/insASAY*^ mice expressed a much lower relative abundance of the *Tbxt*^*Δexon6*^ isoform in the E10.5 embryonic tailbud than that observed in the corresponding in vitro differentiated mouse ES cells modelling primitive streak cells of E6.5 embryos (Fig. 4e, left, and Extended Data Fig. 6c). Although it remains unclear why this difference occurred, it may relate to differential splicing regulation in different cell types^40^. Consequently, the fact that our Alu-pair insertion model did not express high levels of the *Tbxt*^*Δexon6*^ transcript in the embryonic tailbud renders this particular mouse model as inconclusive, beyond the insight that small amounts of this isoform are insufficient to lead to tail loss.

Having noted that the relative abundance of the *Tbxt*^*Δexon6*^ transcript is important for regulating tail length, we next aimed to generate mice with further increased relative abundance of the *Tbxt*^*Δexon6*^ transcript. To do so, we crossed *Tbxt*^*Δexon6/+*^ heterozygous mice with the *Tbxt*^*insRCS2*^ mice. Notably, all 19 compound heterozygous mice (*Tbxt*^*insRCS2/Δexon6*^) presented a complete absence of an external tail (Fig. 4f and Table 2). This phenotype was validated through multiple litters of mice generated from breeding pairs between different *Tbxt*^*Δexon6/+*^ founder lines and *Tbxt*^*insRCS2*^ mice of both heterozygotes (*Tbx*^*insRCS2/+*^) and homozygotes (*Tbxt*^*insRCS2/insRCS2*^) (Table 2). Moreover, *Tbxt*^*insRCS2/Δexon6*^ mice constituted less than the expected 50% among the offspring from breeding *Tbxt*^*Δexon6/+*^ and *Tbxt*^*insRCS2/insRCS2*^ mice pairs, which indicated that some *Tbxt*^*insRCS2/Δexon6*^ embryos may not survive through development. Thus, although the exon 6-deletion heterozygotes (*Tbxt*^*Δexon6/+*^) exhibited incomplete penetrance of tail phenotypes, when crossed with the *Tbxt*^*insRCS2*^ allele, the phenotype was strong, which suggests that production of the tail depends on a minimal abundance of the *Tbxt* full-length isoform. Alternatively, suppression of tail development depends on a higher-than-threshold abundance of *Tbxt*^*Δexon6*^ transcript (Fig. 4f and Table 2). Together, these results demonstrate that the relative abundance of *Tbxt* isoforms is important for regulating tail development.

**Table 2:** Genotype and phenotype analyses of *Tbxt*^*Δexon6*^ and *Tbxt*^*insRCS2*^ breeding results

Breeding type	Offspring genotypes	Total no. of mice examined	Tail phenotypes			
			No tail	Short tail	Kinked tail	Long tail
*Tbxt*^*insRCS2/+*^ × *Tbxt*^*∆exon6/+*^	*Tbxt*^*insRCS2/∆exon6*^	7	7 (2)^a^	0	0	0
	*Tbxt*^*∆exon6/+*^	11	0	0	1	10
	*Tbxt*^*insRCS2/+*^ or *Tbxt*^*+/+*^	12	0	0	0	12
*Tbxt*^*insRCS2/insRCS2*^ × *Tbxt*^*∆exon6/+*^	*Tbxt*^*insRCS2/∆exon6*^	12	12 (4)^a^	0	0	0
	*Tbxt*^*insRCS2/+*^	30	0	0	0	30

Note that tail phenotypes were categorized into no tail, short tail, kinked tail and long tail, as exemplified in Fig. 3e.

^a^Numbers in parentheses indicate 2 mice out of the 7, and 4 out of the 12, were runts and died between 1 and 3 months after birth.

## Homozygous removal of *Tbxt* exon 6 is lethal

**Extended Data Fig. 8:**
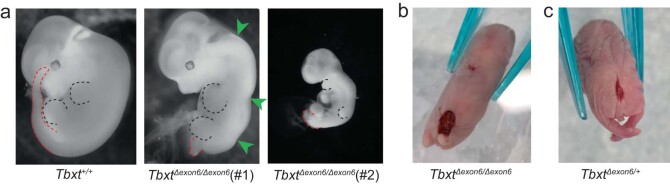
Exon6 deletion of *Tbxt* may lead to neural tube defects in mouse. **a**, Analyzing E11.5 *Tbxt*^*Δexon6*/*Δexon6*^ mouse embryos obtained through intercrossing *Tbxt*^*Δexon6*/+^ mice. *Tbxt*^*Δexon6*/*Δexon6*^ embryos either developed neural tube closure defects (middle) that died at birth or arrested at approximately stage E9 during development (right). Red and black dashed lines mark the embryonic tail regions and limb buds, respectively. Green arrowheads indicate malformed spinal cord regions. **b**-**c**, Both *Tbxt*^*Δexon6*/*Δexon6*^ (**b**) and *Tbxt*^*Δexon6*/*+*^ (**c**) neonatal mice may present neural tube closure defects during embryonic development. The presented embryos were the only two cases found in this study that died after birth with neural tube closure defects.

Our mouse work enabled us to study tail phenotypes in mutant mice across different relative abundance of the *Tbxt* full-length and *Tbxt*^*Δexon6*^ transcripts. We observed a correspondence between mice that expressed a higher abundance of the *Tbxt* full-length transcript with longer tail phenotypes, and mice with short-tail or no-tail phenotypes that expressed a higher abundance of the *Tbxt*^*Δexon6*^ transcript (Fig. 4g). To study the extreme case in which the mice only express *Tbxt*^*Δexon6*^ transcript and not the *Tbxt* full-length transcript, we investigated the developmental phenotypes of *Tbxt*^*Δexon6*/*Δexon6*^ mice through intercrossing experiments. Intercrossing *Tbxt*^*Δexon6/+*^ mice across multiple litters—and replicated in different founder lines—we failed to produce viable homozygotes (Table 1). Dissecting intercrossed stage E11.5 embryos showed that homozygotes either had arrested development at approximately stage E9 or developed with spinal cord malformations that consequently led to death at birth (Extended Data Fig. 8a). Notably, one *Tbxt*^*Δexon6*/*Δexon6*^ pup that died exhibited neural-tube-closure defects similar to the spina bifida condition in humans (Extended Data Fig. 8b). Moreover, a *Tbxt*^*Δexon6*/*+*^ pup that died after birth also exhibited neural-tube-closure defects (Extended Data Fig. 8c). Together, these results indicate that the expression of the *Tbxt*^*Δexon6*^ isoform may induce neural tube defects.

**Extended Data Fig. 9:**
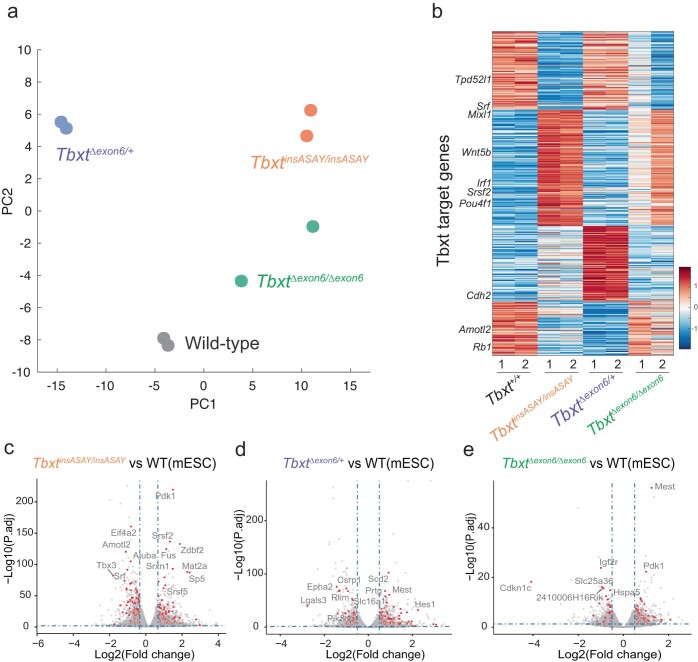
RNA-seq analysis of Tbxt target genes in differentiated mESC lines across genotypes. The analyzed mESC lines include wild-type, *Tbxt*^*insASAY/insASAY*^, *Tbxt*^*Δexon6*/*+*^, and *Tbxt*^*Δexon6*/*Δexon6*^ genotypes, each in duplicates. **a**, Scatter plot of the samples using the first two principal component (PC) coordinates in principal component analysis. **b**, Heatmap of Tbxt-target genes that were differentially expressed across the analyzed samples. Tbxt-target gene list was obtained from Tbxt ChIP-seq results using in vitro differentiated mESCs^41^. Functionally characterized Tbxt-target genes were labeled on the y-axis of the heatmap. **c**–**e**, Volcano plots of differentially expressed (DE) genes comparing mutant mESCs with the wild-type mESC. DE genes were identified using DESeq2 (version 1.40.2)^60^ through its default two-sided Wald test and a cutoff of log2 fold expression change >0.5 and multiple test-adjusted *p* value (*p.adj*) <0.05. For each plot, DE Tbxt-target genes were highlighted in red, and the top DE genes among this group were labeled.

The *Tbxt*^*Δexon6*^ transcript may lead to the production of a shortened transcription factor with limited interactions with other factors or one that exhibits additional functional interactions. To begin to study the effect of this isoform on known *Tbxt* target genes^41^, we analysed the transcriptomes of differentiated mouse ES cell lines from the wild-type, *Tbxt*^*insASAY/insASAY*^, *Tbxt*^*Δexon6*/*+*^ and *Tbxt*^*Δexon6*/*Δexon6*^ genotypes (Extended Data Fig. 9 and Supplementary Data 5). Gene expression of *Tbxt* targets varied across mouse ES cell lines exhibiting different ratios of long and short *Tbxt* isoforms (Extended Data Fig. 9), which indicated a complicated gene regulation network. Additional work will be required to address the possibility that the combination of the two *Tbxt* isoforms leads to new regulatory functionality.

## Discussion

We presented evidence for a plausible evolutionary scenario for tail-loss evolution in hominoids, which involves the insertion of an AluY element into an intron of *TBXT*. As opposed to directly interfering with a splice site, we showed that this element interacts with a simian-shared AluSx1 element in the neighbouring intron, leading to a hominoid-specific AS isoform of *TBXT* (Fig. 1). Experimental deletion of either AluY or its interaction partner AluSx1 eliminated this *TBXT* AS in differentiated human ES cells (Fig. 2). When we engineered the mouse *Tbxt* gene with the human *TBXT* gene structure by inserting the AluSx1–AluY pair—as well as Alu-independent inverted RCSs in a separate mouse ES cell line—we confirmed production of the same exon-skipped splicing isoform (Fig. 4).

The AS mediated by Alu pairing in *TBXT* demonstrates how an interaction between intronic transposable elements can substantially modulate gene function to affect a complex trait. The human genome contains around 1.8 million copies of short interspersed nuclear elements—including about 1 million Alu elements—of which more than 60% are intronic^42^. Systematically searching for such interactions may lead to the identification of additional functional roles by which these elements affect human development and disease. Notably, inverted Alu pairs can facilitate the biogenesis of exonic circular RNAs (circRNAs) through ‘backsplicing’^43,44^. Thus, it is an interesting possibility that the interactions between paired transposable elements might create both functional splice variants and circRNA isoforms from the same genetic locus^45^. Furthermore, our results demonstrated that a completely different (non-Alu) inverted repeat sequence in the introns flanking an exon may also lead to exon skipping. Thus, a global search for such sequence configurations might reveal additional instances of exon skipping caused by this type of sequence configuration.

The main results of our mouse work demonstrated a correspondence between the relative abundance of *Tbxt* isoforms and tail-length phenotypes (Fig. 4g). Expression of the *Tbxt*^*Δexon6*^ transcript in mice—along with the full-length transcript—was sufficient to induce shorter tail to no-tail phenotypes (Fig. 3). Moreover, *Tbxt* AS induced by the intronic RCS pair stably modulated tail length (*Tbxt*^*insRCS2/insRCS2*^ mice; Fig. 4). Finally, we showed that a compound heterozygote with an increased relative abundance of the *Tbxt*^*Δexon6*^ transcript (*Tbxt*^*insRCS2/Δexon6*^ mice) stably exhibits a no-tail phenotype (Fig. 4f,g and Table 2).

Previous studies have shown that the peptide encoded by the exon 6 sequence constitutes part of the transcription regulation domains, but not the DNA-binding domain^9^ (Extended Data Fig. 3e,f). Thus, the AS-induced *TBXT*^*Δexon6*^ transcript may encode for a transcription factor with altered transcription regulation function. Indeed, our transcriptomics analyses of in vitro differentiated mouse ES cells across genotypes found that cells expressing both *Tbxt* isoforms have distinct transcriptome features compared with wild-type cells or cells with *Tbxt*^*exon6*^ homozygous deletion. Notably, this AluY insertion-induced *TBXT*^*Δexon6*^ isoform is different from previously reported mutants of this gene^28,29,38^. Future work is required to reveal the detailed DNA-binding pattern and the transcription regulation functions that the TBXT(Δexon6) isoform protein may play in mediating mesoderm initiation and tail-loss development.

**Extended Data Fig. 10:**
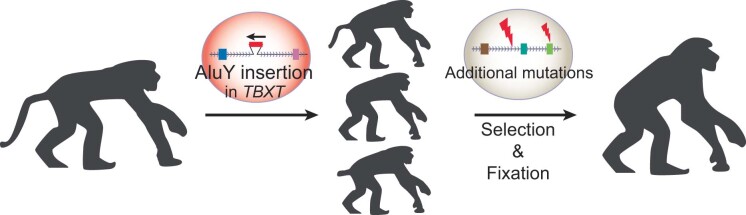
A model for tail-loss evolution in the early hominoids. The AluY insertion in *TBXT* may have marked a key genetic event that contributed to tail-loss evolution in the hominoid common ancestor. Additional genetic changes – pre-existing in the ancestral genome or occurring after AluY insertion – may have also acted to promote or stabilize the no-tail phenotype in the early hominoids.

These results support an inference of how our hominoid ancestors evolved the loss of the tail. In this scenario, AluY insertion either induced the shortening or partial loss of the tail in early hominoid ancestors. However, even if the AluY insertion substantially influenced tail-loss evolution in hominoids, additional genetic changes may have acted to stabilize the no-tail phenotype (Extended Data Fig. 10). Such possible hominoid-specific variants in tail-development-related genes (such as those presented in Supplementary Data 1–4) may have preexisted in the ancestral genome or occurred after the AluY insertion. Such a possible set of genetic events suggest that a change to the AluY element in modern hominoids would be unlikely to result in the reappearance of the tail. Moreover, tail loss or reduction occurred independently multiple times throughout primate evolution, including in loris (Lorisidae), mandrill (*Mandrillus*) and some species of macaques (*Macaca*). As the genome sequences of an increasing number of primates becomes available^46^, it will be interesting to study aspects of convergent evolution involved in the diverse genetic mechanisms that mediated tail-loss evolution.

The specific evolutionary pressures relating to the loss of the tail in hominoids are not clear, although they are probably involved in enhanced locomotion in the transition to a non-arboreal lifestyle. We suggest, however, that the selective advantage must have been strong because the loss of the tail may have included an evolutionary trade-off of neural tube defects, as demonstrated by the presence of neural-tube-closure defects in mice expressing the *Tbxt*^*Δexon6*^ transcript (Extended Data Fig. 8). Notably, mutations leading to neural tube defects and/or sacral agenesis have been detected in the coding and noncoding regions of the *TBXT* gene^47–50^. We therefore speculate that the evolutionary trade-off involving the loss of the tail—made approximately 25 million years ago—may continue to influence human health today.

## Methods

### Comparative genomics analyses of tail-development-related genes

Hominoid evolution represents an extended stage in primate evolution that involved many phenotypic changes and widespread genomic sequence changes. Therefore, querying for hominoid-specific mutations across the genome results in tens of millions of candidates, with most of them disposed in non-coding regions. We used the following criteria to define that a candidate variant may contribute to the tail-loss evolution in hominoids: (1) has to be hominoid-specific, which means that the variant sequence or amino acid is unique to hominoid species and cannot be shared by any other species that have tails; (2) the function of the associated genes relates to tail development. Tail-development-related genes in vertebrates were collected from the MGI phenotype database and additional literature not covered by the MGI database. The initial analyses mainly covered genes extracted from the MGI term MP0003456 for ‘absent tail’ phenotype (https://www.informatics.jax.org/vocab/mp_ontology/MP:0003456), to a total of 31 genes. Additional analyses included genes from MP0002632 for ‘vestigial tail’ (https://www.informatics.jax.org/vocab/mp_ontology/MP:0002632) and MP0003456 for ‘short tail’ (https://www.informatics.jax.org/vocab/mp_ontology/MP:0000592). Together, the final list of genes related to vertebrate tail development included 140 genes (as of MGI updates in February 2023) and the mutations of which are reported to be related to tail-reduction phenotypes (Supplementary Data 1).

Gene structure annotations of the 140 genes were downloaded from BioMart of Ensembl 109 (https://useast.ensembl.org/info/data/biomart/index.html). The longest transcript with the most exons were selected for each gene. Multiz30way alignments of genomic sequences across 27 primate species were downloaded from the UCSC Genome Browser. We selected all six hominoid species (hg38, gorGor5, panTro5, panPan2, ponAbe2 and nomLeu3) to calculate a hominoid-consensus sequence, and used two non-hominoid species (pig-tailed macaque, macNem1, and marmoset, calJac3) as the outgroups. The homologous regions of the 140 genes, together with 10,000 bp both upstream and downstream sequences, in the 8 species were extracted from Multiz30way alignment using bedtools (v.2.30.0)^52^. Hominoid-specific variants were identified using the following parameters: SNVs or substitutions shared by six hominoid species but different in any of the two outgroup monkey species were identified as putative hominoid-specific SNVs (Supplementary Data 2); DNA sequences present in all six hominoid species but absent in either of the two outgroup monkey species were identified as hominoid-specific insertions (Supplementary Data 3); and DNA sequences absent from all six hominoid species but present in both of the two outgroup monkey species were identified as hominoid-specific deletions (Supplementary Data 4). Notably, our criteria for analysing hominoid-specific variants may include a small proportion of false-positive hits that are outgroup-specific variants.

We used the Ensembl variant effect predictor (integrated in Ensembl 109)^53^ to infer the potential functional impact of the detected hominoid-specific SNVs, insertions and deletions. Owing to the lack of an ancestral genome as the reference sequence, variant effect predictor predictions were performed inversely using the human/hominoid genomic sequence as the reference allele, and the outgroup sequence served as the alternative allele. SNVs annotated as either ‘deleterious’ (<0.05) in the SIFT score or ‘damaging’ (>0.446) in the PolyPhen score (53 instances), and insertions (6 instances) or deletions (2 instances) that affect protein sequences were collected for further manual inspection comparison across species. This additional inspection was performed across the Cactus Alignment of the genomes across 241 species in the UCSC Genome Browser Comparative Genomics module^51^. This inspection found that most of the annotated variants that may affect host gene function fell into three categories: (1) outgroup-specific variants; (2) false-positive annotation of the variant function in a minor transcript; and (3) missense variants in hominoid species but sharing the same amino acid in at least one other tailed species. These variants were not considered as candidates that may have affected tail-loss evolution in hominoids. Excluding these variants, we identified nine variants as true hominoid-specific coding region mutations, including seven SNVs and two insertions and deletions (Supplementary Data 1). Following identification of top candidates, protein sequence alignments across representative vertebrate species were downloaded from the NCBI database and analysed using the MUSCLE algorithm with MEGA X software and default settings^54^.

### RNA secondary structure prediction

RNA secondary structure prediction of the human *TBXT* exon 5–intron 5–exon 6–intron 6–exon 7 sequence was performed using RNAfold (v.2.6.0) through the ViennaRNA Web Server (http://rna.tbi.univie.ac.at/)^35^. The algorithm calculates the folding probability using a minimum free energy matrix with default parameters. In addition, the calculation included the partition function and base pairing probability matrix. Notably, human *TBXT* transcripts were annotated to have a 5′ untranslated region exon, making its exon numbers differ from most of other species, including mouse. To simplify, we referred to the first coding exon of human *TBXT* as exon 1 and thus the alternative spliced exon as exon 6, consistent with mouse *Tbxt*. RNA secondary structure prediction used the DNA sequence from exon 5 to exon 7 following this order.

### Human ES cell culture and differentiation

Human ES cells (WA01, also called H1, from WiCell Research Institute) were authenticated by the distributor WiCell using short tandem repeat profiling to authenticate the cell lines. Human ES cells were cultured in feeder-free conditions on tissue-culture-grade plates coated with human ES cell-qualified Geltrex (Gibco, A1413302). Geltrex was 1:100 diluted in DMEM/F-12 (Gibco, 11320033) supplemented with 1× Glutamax (100X, Gibco, 35050061) and 1% penicillin–streptomycin (Gibco, 15070063). Before seeding human ES cells, the plate was treated with Geltrex working solution in a tissue culture incubator (37 °C and 5% CO_2_) for at least 1 h.

StemFlex medium (Gibco, A3349401) was used for human ES cell maintenance and culturing in a feeder-free condition according to the manufacturer’s protocol. In brief, StemFlex complete medium was made by combining StemFlex basal medium (450 ml) with 50 ml of StemFlex supplement (10×) plus 1% penicillin–streptomycin. Each Geltrex-coated well on a 6-well plate was seeded with 200,000 cells to obtain about 80% confluence in 3–4 days. Human ES cells were cryopreserved in PSC Cryomedium (Gibco, A2644601). The culture medium was supplemented with 1× RevitaCell (100×, Gibco, A2644501, which is also included in the PSC Cryomedium kit) when cells had gone through stressed conditions, such as freezing-and-thawing or nucleofection. RevitaCell supplemented medium was replaced with regular StemFlex complete medium on the second day. Human ES cells grown under the RevitaCell condition might become stretched but would recover after returning to the normal StemFlex complete medium. All human ES cell lines tested negative during our routine quantitative PCR-based mycoplasma tests.

The human ES cell differentiation assay to induce a gene expression pattern of the primitive streak state was adapted from a previously published method^36^. On day −1, freshly cultured human ES cell colonies were dissociated into clumps (2–5 cells) using Versene buffer (with EDTA, Gibco, 15040066). The dissociated cells were seeded on Geltrex-coated 6-well tissue culture plates at 25,000 cells per cm^2^ (0.25 M per well in the 6-well plates) in StemFlex complete medium. Differentiation to the primitive streak state was initiated on the next day (day 0) by switching StemFlex complete medium to basal differentiation medium. Basal differentiation medium (50 ml) was made using 48.5 ml DMEM/F-12, 1% Glutamax (500 µl), 1% ITS-G (500 µl, Gibco, 41400045) and 1% penicillin–streptomycin (500 µl), and supplemented with 3 µM GSK3 inhibitor CHIR99021 (10 µl of 15 mM stock solution in DMSO; Tocris, 4423). The cells were collected at differentiation day 1 to 3 for downstream experiments, which confirmed the expression fluctuations of mesoderm genes (*TBXT* and *MIXL1*) in a 3-day differentiation period^36^ (Extended Data Fig. 3).

### Mouse ES cell culture and differentiation

The mouse ES cell line (MK6) derived from the C57BL/6J mouse strain was obtained from the NYU Langone Health Rodent Genetic Engineering Laboratory. The wild-type MK6 mouse ES cell line was authenticated by its competence for contributing to embryos when cultured on feeder-cell-dependent conditions followed by blastomere injection. MK6 mouse ES cells used in this study were cultured in both feeder-dependent and feeder-free culture conditions depending on the purposes of the experiment. All mouse ES cell lines tested negative during our routine quantitative PCR-based mycoplasma tests. For feeder-dependent mouse ES cell culture conditions, mouse ES cells were plated on a pre-seeded monolayer of mouse embryonic fibroblast (MEF) cells (CellBiolabs, CBA-310). MEF-coated plates were prepared by seeding 50,000 cells per cm^2^ in tissue culture plates treated with 0.1% gelatin solution (EMD Millipore, ES-006-B). MEF culturing medium was made from DMEM (Gibco, 11965118) with 10% FBS (GeminiBio, 100–500), 0.1 mM MEM non-essential amino acids (Gibco, 11140050), 1× Glutamax (Gibco, 35050061) and 1% penicillin–streptomycin (Gibco, 15070063). Mouse ES cell medium was made from Knockout DMEM (Gibco, 10829018) containing 15% (v/v) FBS (Hyclone, SH30070.03), 0.1 mM β-mercaptoethanol (Gibco, 31350010), 1× MEM non-essential amino acids (Gibco, 11140050), 1× Glutamax (Gibco, 35050061), 1× nucleosides (Millipore, ES-008-D) and 1,000 units ml^–1^ LIF (EMD Millipore, ESG1107).

For feeder-free mouse ES cell culture conditions, cells were grown on tissue-culture-grade plates that were pre-coated with mouse ES cell-qualified 0.1% gelatin (EMD Millipore, ES-006-B) at room temperature for at least 30 min. Before seeding mouse ES cells, feeder-free mouse ES cell culturing medium was added to a gelatin-treated plate and warmed in a 37 °C and 5% CO_2_ incubator for at least 30 min. Feeder-free mouse ES cell culturing medium, also called ‘80/20’ medium, comprises 80% 2i medium and 20% of the above-mentioned mouse ES cell medium by volume. The 2i medium was made from a 1:1 mix of Advanced DMEM/F-12 (Gibco, 12634010) and Neurobasal-A (Gibco, 10888022), followed by adding 1× N2 supplement (Gibco, 17502048), 1× B-27 supplement (Gibco, 17504044), 1× Glutamax (Gibco, 35050061), 0.1 mM β-mercaptoethanol (Gibco, 31350010), 1,000 units ml^–1^ LIF (Millipore, ESG1107), 1 µM MEK1/2 inhibitor (Stemgent, PD0325901) and 3 µM GSK3 inhibitor CHIR99021 (Tocris, 4423).

The mouse ES cell differentiation protocol for inducing *Tbxt* gene expression was adapted from a previously described method in a feeder cell-free condition^37^. Cells were first plated in 80/20 medium for 24 h on a gelatin-coated 6-well plate, followed by switching to N2/B27 medium without LIF or 2i for another 2 days of culture. The N2/B27 medium (50 ml) was made with 18 ml Advanced DMEM/F-12, 18 ml Neurobasal-A, 9 ml Knockout DMEM, 2.5 ml Knockout Serum Replacement (Gibco, 10828028), 0.5 ml N2 supplement, 1 ml B27 supplement, 0.5 ml Glutamax (100×), 0.5 ml nucleosides (100×) and 0.1 mM β-mercaptoethanol. Then the N2/B27 medium was supplemented with 3 µM GSK3 inhibitor CHIR99021 to induce differentiation (day 0). The cells were collected at differentiation day–3 for downstream experiments, which showed consistent results of *Tbxt* gene expression fluctuations in a 3-day differentiation period.

### CRISPR targeting

All guide RNAs of the CRISPR experiments were designed using the CRISPOR algorithm integrated in the UCSC Genome Browser^55^. Guide RNAs were cloned into the pX459V2.0-HypaCas9 plasmid (AddGene, plasmid 62988) or its custom derivative by replacing the puromycin-resistance gene with the blasticidin-resistance gene. Guide RNAs in this study were designed in pairs to delete the intervening sequences. Insertion sites for the AluSx1 and AluY pair in mouse *Tbxt* (*Tbxt*^*insASAY*^) were selected by the guide RNA quality and the relative distance compared to the human *TBXT* gene structure. The insertion site for the RCS element (*Tbxt*^*insRCS*^) was the same as for insertion of the AluY element. The CRISPR-targeting sites and guide RNA sequences are listed in Supplementary Table 2.

All oligonucleotides (plus Golden-Gate assembly overhangs) were synthesized by Integrated DNA Technologies (IDT) and ligated into an empty pX459V2.0 vector following the standard Golden Gate Assembly protocol using BbsI restriction enzyme (NEB, R3539). The constructed plasmids were purified from 3 ml *Escherichia coli* cultures using a ZR Plasmid MiniPrep Purification kit (Zymo Research, D4015) for sequence verification. Plasmids for delivering into ES cells were purified from 250 ml *E.* *coli* cultures using a PureLink HiPure Plasmid Midiprep kit (Invitrogen, K210005). To facilitate DNA delivery to ES cells through nucleofection, the purified plasmids were resolved in Tris-EDTA buffer (pH 7.5) to a concentration of at least 1 µg µl^–1^ in a sterile hood.

### DNA delivery

DNA delivery into human or mouse ES cells for CRISPR–Cas9 targeting was performed using a Nucleofector 2b device (Lonza, BioAAB-1001). A Human Stem Cell Nucleofector kit 1 (VPH-5012) and a mouse ES cell Nucleofector kit (Lonza, VVPH-1001) were used for delivering DNA into human and mouse ES cells, respectively. ES cells were double-fed the day before the nucleofection experiment to maintain a superior condition.

Before performing nucleofection on human ES cells, 6-cm tissue culture plates were treated with 0.5 µg cm^–2^ rLaminin-521 in a 37 °C and 5% CO_2_ incubator for at least 2 h. rLaminin-521-treated plates provide the best viability when seeding human ES cells as single cells. Cultured human ES cells were washed with DPBS and dissociated into single cells using TrypLE Select Enzyme (no phenol red; Gibco, 12563011). One million human ES cell single cells were nucleofected using program A-023 according to the manufacturer’s instructions for the Nucleofector 2b device. Transfected cells were transferred onto the rLaminin-521-treated 6-cm plates with pre-warmed StemFlex complete medium supplemented with 1× RevitaCell but not penicillin–streptomycin. Antibiotic selection was performed 24 h after nucleofection with puromycin (final concentration of 0.8 μg ml^–1^; Gibco, A1113802).

Mouse ES cells were dissociated into single cells using StemPro Accutase (Gibco, A1110501), and 5 million cells were transfected using program A-023 according to the manufacturer’s instructions. Exon 6 deletion in mouse ES cells was performed using cells cultured in the feeder-free condition. Nucleofected cells were plated on 0.1% gelatin-treated 10-cm plates, followed by antibiotic selection 24 h after nucleofection with blasticidin (final concentration of 7.5 µg ml^–1^; Gibco, A1113903). The insertion of the *Alu*Sx1–AluY pair and insRCS engineering were performed using mouse ES cells cultured on a feeder-dependent condition. Mouse ES cells were plated on a monolayer of MEF cells seeded on 0.1% gelatin-treated 10-cm plates, followed by antibiotic selection 24 h after nucleofection.

Together with the pX459V2.0-HypaCas9-gRNA plasmids for nucleofection, single-strand DNA oligonucleotides were co-delivered for microhomology-induced deletion of the targeted sequences^56^. These ssDNA sequences were synthesized by IDT through its Ultramer DNA Oligo service, including three phosphorothioate bond modifications on each end. Detailed sequence information of these long ssDNA oligonucleotides are listed in Supplementary Table 3.

For *Tbxt*^*insASAY*^ and *Tbxt*^*insRCS*^ engineering, homology-based repairing template plasmids, including a selection marker gene *puro*-*ΔTK*, (puromycin-resistance gene for positive selection and *ΔTK*, a truncated version of herpes simplex virus type 1 thymidine kinase, for negative selection, as presented in Extended Data Fig. 7), was transfected together with the pX459V2.0-HypaCas9-gRNA plasmids. Following nucleofection and antibiotic selection (0.8 μg ml^–1^ puromycin for 3 days starting from the second day of nucleofection), single clones were picked, followed by PCR genotyping of CRISPR–Cas9-targeted loci (exon 6 deletion, inserting AluY, inserting AluSx1 or inserting RCS). The genotyping PCR primers are listed in Supplementary Table 4.

PCR genotyping-confirmed clones were further validated using Capture-seq (see below) to confirm the genotype and to exclude the possibility of any random integration of plasmid DNA. Subsequently, Cre recombinase was transiently introduced to remove the selection marker *puro*-*ΔTK*. Cells were treated with 250 nM ganciclovir for counter-selecting *ΔTK*-negative cells as the selection marker gene-depleted cells. Following isolation of single mouse ES cell clones of *Tbxt*^*insASAY*^ and *Tbxt*^*insRCS*^ mouse ES cells without the selection marker gene, these clones were used for downstream experiments, including in vitro differentiation assays and blastocyst injection for generating mouse models.

### Capture-seq genotyping

Capture-seq, or targeted sequencing of the loci of interest, was performed as previously described^39^. Conceptually, Capture-seq uses custom biotinylated probes to pull-down the sequences at genomic loci of interest from the standard whole-genome sequencing libraries, thereby enabling sequencing of the specific genomic loci in a much higher depth while reducing the cost.

Genomic DNA was purified from mouse ES cells or from ear punches of mice of interest using a Zymo Quick-DNA Miniprep Plus kit (D4068) according to the manufacturer’s protocol. DNA sequencing libraries compatible for Illumina sequencers were prepared following the standard protocol. In brief, 1 µg of gDNA was sheared to 500–900 base pairs in a 96-well microplate using a Covaris LE220 (450 W, 10% duty factor, 200 cycles per burst and 90-s treatment time), followed by purification with a DNA Clean and Concentrate-5 kit (Zymo Research, D4013). Sheared and purified DNA were then treated with end repair enzyme mix (T4 DNA polymerase, Klenow DNA polymerase and T4 polynucleotide kinase, NEB, M0203, M0210 and M0201, respectively), and A-tailed using Klenow 3′−5′ exo-enzyme (NEB, M0212). Illumina sequencing library adapters were subsequently ligated to DNA ends followed by PCR amplification with KAPA 2X Hi-Fi Hotstart Readymix (Roche, KR0370).

Custom biotinylated probes were prepared as bait through nick translation using BAC DNA and/or plasmids as the template. The probes were prepared to comprehensively cover the entire locus. We used BAC lines RP24-88H3 and RP23-159G7, purchased from BACPAC Genomics, to generate bait probes covering the mouse *Tbxt* locus and about 200 kb flanking sequences in both upstream and downstream regions. The pooled whole-genome sequencing libraries were hybridized with the biotinylated baits in solution and purified using streptavidin-coated magnetic beads. Following pull-down, DNA sequencing libraries were quantified using a Qubit 3.0 Fluorometer (Invitrogen, Q33216) with a dsDNA HS Assay kit (Invitrogen, Q32851). The sequencing libraries were subsequently sequenced on an Illumina NextSeq 500 sequencer in paired-end mode.

Sequencing results were demultiplexed using Illumina bcl2fastq (v.2.20), requiring a perfect match to indexing BC sequences. Low-quality reads or bases and Illumina adapter sequences were trimmed using Trimmomatic (v.0.39). Reads were then mapped to the mouse genome (mm10) using bwa (v.0.7.17). The coverage and mutations in and around the *Tbxt* locus were checked through visualization in a mirror version of the UCSC Genome Browser.

### Mouse experiments and generating *Tbxt*^*Δexon6/+*^ mice

All mouse experiments were performed following NYULH’s animal protocol guidelines and performed at the NYU Langone Health Rodent Genetic Engineering Laboratory. Mice were housed in the NYU Langone Health BSL1 barrier facility in a 12-h light to 12-h dark cycle, with ambient temperature and humidity conditions. All experimental procedures were approved by the Institutional Animal Care and Use Committee at NYU Langone Health. Wild-type C57BL/6J (strain 000664) mice were obtained from The Jackson Laboratory.

The *Tbxt*^*Δexon6/+*^ heterozygous mouse model was generated through zygotic microinjection of CRISPR reagents into wild-type C57BL/6J zygotes (Jackson Laboratory strain 000664), adapting a previously published protocol^57^. In brief, *Cas9* mRNA (MilliporeSigma, CAS9MRNA), synthetic guide RNAs and single-stranded DNA oligonucleotide were co-injected into 1-cell stage zygotes following the described procedures^57^. Synthetic guide RNAs were ordered from Synthego as their custom CRISPRevolution sgRNA EZ kit, with the same targeting sites as used in the CRISPR deletion experiment of mouse ES cells (AUUUCGGUUCUGCAGACCGG and CAAGAUGCUGGUUGAACCAG). The co-injected single-stranded DNA oligonucleotide is the same as described above. Injected embryos were then in vitro cultured to the blastomeric stage, followed by embryo transferring to the pseudopregnant foster mothers. Following zygotic microinjection and transferring, founder pups were screened based on their abnormal tail phenotypes. DNA samples were collected through ear punches at about day 21 for PCR genotyping and Capture-seq validation to exclude off-targeting at the *Tbxt* locus.

After confirming the genotype, *Tbxt*^*Δexon6/+*^ founder mice were backcrossed with wild-type C57B/6J mice for generating heterozygous F_1_ mice. Owing to the varied tail phenotypes, intercrossing between F_1_ heterozygotes were performed in two categories: type 1 intercrossing included at least one parent having no tail or a short tail, whereas type 2 intercrossing were mated between two long-tailed F_1_ heterozygotes (Table 1). As summarized in Table 1, both types of intercrossing produced heterogeneous tail phenotypes in F_2_ *Tbxt*^*Δexon6/+*^ mice, thereby confirming the incomplete penetrance of tail phenotypes and the absence of homozygotes (*Tbxt*^*Δexon6/Δexon6*^). Adult mice (>12 weeks) were anaesthetized for X-ray imaging of vertebra using a Bruker In-Vivo Xtreme IVIS imaging system. To confirm the embryonic phenotypes in homozygotes, embryos were dissected at E11.5 gestation stage from the timed pregnant mice using a standard protocol.

### Generating *Tbxt*^*insASAY*^ and *Tbxt*^*insRCS2*^ mice

The engineered *Tbxt*^*insASAY*^ and *Tbxt*^*insRCS*^ mouse ES cells were injected into either C57BL/6J-albino (Charles River Laboratories, strain 493) blastocysts for chimeric F_0_ founder mice or injected into B6D2F1/J (a F_1_ hybrid between C57BL/6J female and DBA/2J male, Jackson Laboratory strain 100006) tetraploid blastocysts for homozygote F_0_ founder mice production. The tetraploid complementation strategy aimed to generate homozygous mice with the proposed genotype in the F_0_ generation^58^. Through multiple trials of injection using both mouse ES cell lines, we achieved only one *Tbxt*^*insASAY/insASAY*^ F_0_ founder mouse (male) but none for the *Tbxt*^*insRCS*^ mouse. However, during genotype screening for *Tbxt*^*Δexon6/+*^ founder mice, we serendipitously identified a male grey mouse that incorporated a heterozygous insertion in intron 5. Genotype analysis revealed that the insertion was a 220 bp DNA sequence from intron 6 of *Tbxt* (chromosome 17: 8439335–8439554, mm10), inserted in a reverse complementary scenario into intron 5 at a designed CRISPR targeting site (chromosome 17: 8438386, mm10). The inserted sequence insRCS2 in intron 5 therefore forms a 220 bp inverted complementary sequence pair with its original sequence in intron 6 (chromosome 17: 8439335–8439554, mm10), resembling the designed *Tbxt*^*insRCS*^ and *Tbxt*^*insASAY*^ gene structures. This genotype was therefore called *Tbxt*^*insRCS2*^. Capture-seq genotyping of this *Tbxt*^*insRCS2/+*^ mouse confirmed that the *Tbxt*^*insRCS2*^ allele is in the C57BL/6 background, whereas the wild-type *Tbxt* locus of the *Tbxt*^*insRCS2/+*^ founder mouse is from the DBA/2J background. This *Tbxt*^*insRCS2/+*^ mouse was therefore backcrossed to C57BL/6 wild-type mice and further intercrossed between F_1_ heterozygotes to produce homozygotes (*Tbxt*^*insRCS2/insRCS2*^) in the F_2_ generation. Capture-seq analysis of *Tbxt*^*insRCS2/insRCS2*^ mice confirmed their C57BL/6 background at the *Tbxt* locus (Extended Data Fig. 8). We also compared the tail phenotypes in age-matched C57BL/6 and DBA/2J mice and found no difference (data not shown), which suggested that any genetic background difference between the two strains does not affect tail length. The *Tbxt*^*insRCS2*^ mice (both heterozygotes and homozygotes) were therefore used for the analysis of tail phenotypes.

The *Tbxt*^*insASAY*^ and *Tbxt*^*insRCS2*^ founder mice, both male, were separately backcrossed to wild-type C57B/6J mice for generating heterozygous F_1_ pups, followed by intercrossing between F_1_ heterozygotes to generate homozygotes in F_2_ generation. With all genotypes available, mouse tail lengths were measured monthly across genotypes and sex groups. Additionally, two types of breading pairs, *Tbxt*^*insRCS2/+*^ × *Tbxt*^*Δexon6/+*^ and *Tbxt*^*insRCS2/insRCS2*^ × *Tbxt*^*Δexon6/+*^, were performed across different founder lines of *Tbxt*^*Δexon6/+*^ mice to analyse tail phenotypes in their offspring. These results are summarized and presented in Table 2.

To analyse the isoform expression patterns of mouse *Tbxt* in the embryonic tailbud region, wild-type, heterozygote and homozygote embryos from intercrossing experiments (*Tbxt*^*insRCS2/+*^ × *Tbxt*^*insRCS2/+*^, *Tbxt*^*insASAY/+*^ × *Tbxt*^*insASAY/+*^) were dissected at the E10.5 gestation stage. The tailbud for each embryo was collected for isolating total RNA, and together with embryonic tissue for gDNA to be used for genotyping. These results are presented in Fig. 4e.

### Splicing isoform detection

Total RNA was collected from undifferentiated and differentiated cells of both human and mouse ES cells, and from embryonic tailbud samples, using a standard column-based purification kit (Qiagen RNeasy Kit, 74004). DNase treatment was applied during RNA extraction to remove any potential DNA contamination. Following extraction, RNA quality was assessed through electrophoresis based on ribosomal RNA integrity. Reverse transcription was performed using 1 µg of high-quality total RNA for each sample and a High-Capacity RNA-to-cDNA kit (Applied Biosystems, 4387406). DNA oligonucleotides used for RT–PCR and/or quantitative RT–PCR are listed in Supplementary Table 1.

### Transcriptomics analyses in differentiated mouse ES cells

Total RNA samples isolated from day-1 in vitro-differentiated mouse ES cell lines across wild-type, *Tbxt*^*insASAY/insASAY*^, *Tbxt*^*Δexon6*/*+*^ and *Tbxt*^*Δexon6*/*Δexon6*^ genotypes were used for bulk RNA sequencing analysis. RNA samples were prepared using a standard column-based purification kit (Qiagen RNeasy kit, 74004). Two biological replicates were prepared for each mouse ES cell genotype, with the two *Tbxt*^*Δexon6*/*Δexon6*^ mouse ES cell samples coming from different clones. RNA sequencing libraries were prepared using a NEBNext Ultra II Directional RNA Library Prep kit (NEB, E7765L) through its polyA mRNA sequencing workflow by using the NEBNext Poly(A) mRNA Magnetic Isolation Module (NEB, E7490L).

Raw sequencing reads were mapped to the mouse genome (mm10) with STAR (v.2.7.2a) aligner^59^. The resultant strand-specific read counts of all samples were integrated into a matrix for downstream analysis. Differentially expressed genes were detected using DESeq2 (v.1.40.2)^60^, using its default two-sided Wald test with the cut-off of log_2_(fold expression change) > 0.5 and multiple test-adjusted *P* value < 0.05. The top 500 variable genes from DESeq2 across all samples were used to perform principal component analysis. The *Tbxt* target genes were obtained from a previous publication^41^, defined by significant *Tbxt* ChIP–seq peak signals detected in in vitro-differentiated mouse ES cells. The set of *Tbxt* target genes was intersected with the significant differentially expressed genes identified in each mutant sample compared with the wild-type controls, and these were aggregated to generate the overall set of differentially expressed *Tbxt* target genes across the analysed mouse ES cell lines. These differentially expressed *Tbxt* target genes were visualized using a heatmap, with the log_10_-transformed normalized transcript matrix followed by *z* score standardization across samples.

### Reporting summary

Further information on research design is available in the Nature Portfolio Reporting Summary linked to this article.

## Online content

Any methods, additional references, Nature Portfolio reporting summaries, source data, extended data, supplementary information, acknowledgements, peer review information; details of author contributions and competing interests; and statements of data and code availability are available at 10.1038/s41586-024-07095-8.

### Supplementary information

Supplementary Tables This file contains Supplementary Tables 1–4.

Reporting Summary

Supplementary Data 1 Summary of hominoid-specific variants in tail-development-related genes. The file includes a list of genes with documented mouse mutants presenting tail phenotypes in the MGI database (Methods).

Supplementary Data 2 Hominoid-specific SNVs detected in the 140 genes.

Supplementary Data 3 Hominoid-specific insertions detected in the 140 genes.

Supplementary Data 4 Hominoid-specific deletions detected in the 140 genes.

Supplementary Data 5 Processed RNA sequencing results of in vitro-differentiated mouse ES cell lines.

## Data Availability

Raw and processed sequencing data in the manuscript have been deposited into the Gene Expression Omnibus database under accession number GSE252279.

## Code availability

The relevant code and processed data for this manuscript are available from GitHub (https://github.com/boxialaboratory/Tail-Loss-Primates).
