# Supplementary material for: On the genetic basis of tail-loss evolution in humans and apes
Source: Nature. 2024 Feb 28;626(8001):1042–8. doi: 10.1038/s41586-024-07095-8 (PMC10901737; doi:10.1038/s41586-024-07095-8)
Supplement: Supplementary file 1 — This file contains Supplementary Tables 1–4. [file 41586_2024_7095_MOESM1_ESM.pdf]

nature portfolio

https://doi.org/10.1038/s41586-024-07095-8

Supplementary information

On the genetic basis of tail-loss evolution in humans and apes

In the format provided by the authors and unedited

Supplementary Tables

Supplementary Table 1. RT-PCR primer sequences.

RT-PCR Target sites	Orientation	Sequence
Human *TBXT*: exon 8	F	CCTCATAGCCTCATGGACACCTG
	R	TCTTAACCTGAGACTGCCACTGG
Human *TBXT*: exon3-8	F	GGTGACTGCTTATCAGAACGAGGAG
	R	TACTGAGGCTGCATTTCCTTCTTAACC
Human *TBXT*: exon4-7	F	CAGAACGAGGAGATCACAGCTC
	R	GGTACTGACTGGAGCTGGTAGG
Human *MIXL1*	F	GGCGTCAGAGTGGGAAATCC
	R	GGCAGGCAGTTCACATCTACC
Human *ACTB1*	F	CACCATTGGCAATGAGCGGTTC
	R	AGGTCTTTGCGGATGTCCACGT
Mouse *Tbxt*: exon4-7	F	CCAGAATGAGGAGATTACAGCCCT
	R	GGATACTGGCTAGAGCCAGTAGG
Mouse *Actb1*	F	CATTGCTGACAGGATGCAGAAGG
	R	TGCTGGAAGGTGGACAGTGAGG

Supplementary Table 2. CRISPR targeting sites and guide RNA sequences.

Targeting purposes	PAM	Guide RNA sequence	Locus (hg38/mm10)
Delete *Alu*Y in human *TBXT*	TGG	AGACTGTGCCCACTCTCGGG	chr6:166161988-166162010
	TGG	GATAGACCATAAAGATCCCC	chr6:166161557-166161579
Delete *Alu*Sx1 in human *TBXT*	GGG	CACAGTAGTTGTCCCGCTAG	chr6:166163730-166163752
	AGG	GAATGGGGGGAGCTTAAACC	chr6:166163290-166163312
Delete *Tbxt*-exon6 in mouse *Tbxt*	TGG	ATTTCGGTTCTGCAGACCGG	chr17:8438362-8438384
	GGG	CAAGATGCTGGTTGAACCAG	chr17:8438920-8438942
Insert *Alu*Sx1 into mouse *Tbxt* intron 5	GGG	CTACCCGATGAGCCACAATA	chr17:8437898-8437920
Insert *Alu*Y/RCS into mouse *Tbxt* intron 6	TGG	TAGAACTCAAGCCCTTAGTG	chr17:8439161-8439183

Supplementary Table 3. Long DNA oligos used in CRISPR/Cas9-mediated DNA deletion experiments. Target sites correspond to the CRISPR targeting purposes indicted in Supplementary Table 1.

Delete *Alu*Y in human *TBXT*	A*C*T*ACACCAGTGATTCTCCAAATACGGTGCCCAGGCCAGAGCCTCAGCACCACCC|CCCTGGCCTAATTCTCTCTATAACACTGTATATGTCTAGACTTAATTTTGTC*T*G*G
Delete *Alu*Sx1 in human *TBXT*	C*A*G*TGCTGCCTGGAGAATTGTTAGTAGTTTGGAAATTGAAGCCACAGTAGTTGTCCCGC|ACCAGGAGAGTGAGCAGTAAAAGGGTCTACCCCCAGCTAGGAAGGCACCTCCCGTC*T*C*T
Delete *Tbxt*-exon6 in mouse *Tbxt*	T*T*T*ATTCTAGAGCCCATTAACATATCACTCCTGCTCACTTGGTAGAAAGCCACCG|CAGGGGTCCCCAAGGAGGCTTTCATTTCAATATCCATGTGCCTCAGAACATG*C*C*C

Note: “|” indicates a junction site; “*” indicates phosphorothioate bond modification sites.

Supplementary Table 4. Genotyping PCR primer sequences.

**PCR Target sites**	**Orientation**	**Sequence**
Delete human *TBXT-Alu*Y: read through	F	CAGCCAGGCTCAAGAATTCC
	R	GACTTCCTAACCCAATAAGGTCC
Delete human *TBXT-Alu*Y: left junction	F	CAGCCAGGCTCAAGAATTCC
	R	GTGTTCCTAATATTGGAGCATGC
Delete human *TBXT-Alu*Sx1: read through	F	TCCTAGGCTGATTGAACAACCAG
	R	CAAGGCAGGTGAGCTTTCC
Delete human *TBXT-Alu*Sx1: left junction	F	TCCTAGGCTGATTGAACAACCAG
	R	TTAAGCTCCCCCCATTC
Delete mouse *Tbxt-exon6*: read through	F	GCAGTCTGAGTCCTACCTGTG
	R	TGTCAGTCTGGTTCTACACCTGGAGAGTCTTTGATC
Delete mouse *Tbxt-exon6*: left junction	F	GCAGTCTGAGTCCTACCTGTG
	R	GTACAGGACCTACTTGGAGAGC
Delete mouse *Tbxt-exon6*: right junction	F	GACAGGACTGAGTCTCAAGC
	R	TGTCAGTCTGGTTCTACACCTGGAGAGTCTTTGATC
Insert *Alu*Sx1 in mouse *Tbxt*: read through	F	CTCTGTCCCTAAACAATGATCAGG
	R	GACTACCCGATACAATACTTGAGAC
Insert *Alu*Y/RCS in mouse *Tbxt*: read through	F	TGGAGCCGTGGTTTGATGAAC
	R	CAGACAAAGGTTGGTCTAAACAGAC
