# Supplementary material for: On the genetic basis of tail-loss evolution in humans and apes
Source: Nature. 2024 Feb 28;626(8001):1042–8. doi: 10.1038/s41586-024-07095-8 (PMC10901737; doi:10.1038/s41586-024-07095-8)
Supplement: Supplementary file 2 — Reporting Summary [file 41586_2024_7095_MOESM2_ESM.pdf]

nature portfolio

Corresponding author(s): Bo Xia
Jef Boeke
Itai Yanai

Last updated by author(s): Dec 28, 2023

Reporting Summary

Nature Portfolio wishes to improve the reproducibility of the work that we publish. This form provides structure for consistency and transparency in reporting. For further information on Nature Portfolio policies, see our Editorial Policies and the Editorial Policy Checklist.

Statistics

For all statistical analyses, confirm that the following items are present in the figure legend, table legend, main text, or Methods section.

n/a	Confirmed	
☐	☒	The exact sample size (n) for each experimental group/condition, given as a discrete number and unit of measurement
☐	☒	A statement on whether measurements were taken from distinct samples or whether the same sample was measured repeatedly
☒	☐	The statistical test(s) used AND whether they are one- or two-sided<br>*Only common tests should be described solely by name; describe more complex techniques in the Methods section.*
☒	☐	A description of all covariates tested
☒	☐	A description of any assumptions or corrections, such as tests of normality and adjustment for multiple comparisons
☐	☒	A full description of the statistical parameters including central tendency (e.g. means) or other basic estimates (e.g. regression coefficient) AND variation (e.g. standard deviation) or associated estimates of uncertainty (e.g. confidence intervals)
☐	☒	For null hypothesis testing, the test statistic (e.g. F, t, r) with confidence intervals, effect sizes, degrees of freedom and P value noted<br>*Give P values as exact values whenever suitable.*
☒	☐	For Bayesian analysis, information on the choice of priors and Markov chain Monte Carlo settings
☒	☐	For hierarchical and complex designs, identification of the appropriate level for tests and full reporting of outcomes
☒	☐	Estimates of effect sizes (e.g. Cohen's d, Pearson's r), indicating how they were calculated

Our web collection on statistics for biologists contains articles on many of the points above.

Software and code

Policy information about availability of computer code

Data collection	Tail development-related genes in vertebrates were collected from the MGI mouse phenotype database. The gene structure annotations of the 140 genes were downloaded from BioMart of Ensembl 109. Multiz30way alignments of genomic sequences across 27 primate species were downloaded from the UCSC Genome Browser, referring to hg38. The homologous regions of the tail-development genes and their 10Kb upstream and downstream sequences were extracted from Multiz30way alignment using bedtools (v2.30.0). The Tbxt-target genes were collected from Lolas et al. 2014 (citation 41). All software information were described in the Methods section.
Data analysis	We used custom analysis pipeline to extract the primate multiple sequence alignment files at the genomic regions of the designated genes. We used the Variant Effect Predictor (VEP), integrated in Ensembl 109, to infer the potential functional impact. Protein sequence alignment were done by the MUSCLE algorithm using MEGA X software with default settings. RNA secondary structure prediction was performed using RNAfold (version 2.6.0) through the ViennaRNA Web Server (http://rna.tbi.univie.ac.at/). All data analysis using public softwares and/or recourses were described in the Methods section. The relevant code and processed data for this manuscript is available on GitHub (https://github.com/boxialaboratory/Tail-Loss-Primates)

For manuscripts utilizing custom algorithms or software that are central to the research but not yet described in published literature, software must be made available to editors and reviewers. We strongly encourage code deposition in a community repository (e.g. GitHub). See the Nature Portfolio guidelines for submitting code & software for further information.

Data

Policy information about availability of data

All manuscripts must include a data availability statement. This statement should provide the following information, where applicable:

- Accession codes, unique identifiers, or web links for publicly available datasets
- A description of any restrictions on data availability
- For clinical datasets or third party data, please ensure that the statement adheres to our policy

Raw and processed sequencing data in the manuscript has been distributed to Gene Expression Omnibus (GEO) under accession number GSE252196.

Field-specific reporting

Please select the one below that is the best fit for your research. If you are not sure, read the appropriate sections before making your selection.

☒ Life sciences ☐ Behavioural & social sciences ☐ Ecological, evolutionary & environmental sciences

For a reference copy of the document with all sections, see nature.com/documents/nr-reporting-summary-flat.pdf

Life sciences study design

All studies must disclose on these points even when the disclosure is negative.

Sample size	Sample size was not specifically predetermined for the in vitro cell culture experiments. We used independent biological replicates of sample size >=2 and each with at least technical duplicates for in vitro molecular experiments. For mouse mutants analysis, the number of analyzed mice were indicated in the manuscript.
Data exclusions	Tail length measurement in Tbxt-insASAY/insASAY mice excluded one mouse which has a shorter tail but obviously due to injury.
Replication	The molecular experiments for analyzing TBXT splicing in ESCs had been replicated independently for >=3 times. All attempts of the replication experiments were consistent with the reported results. The incomplete penetrance of mouse mutant phenotypes was stable.
Randomization	The mouse experiments and tail length measurement were obtained randomly across multiple litters.
Blinding	The Investigators were not blinded to the mouse experiments as the results were consistent across multiple researchers in pilot experiments.

Reporting for specific materials, systems and methods

We require information from authors about some types of materials, experimental systems and methods used in many studies. Here, indicate whether each material, system or method listed is relevant to your study. If you are not sure if a list item applies to your research, read the appropriate section before selecting a response.

Materials & experimental systems

n/a	Involved in the study	
☒	☐	Antibodies
☐	☒	Eukaryotic cell lines
☒	☐	Palaeontology and archaeology
☐	☒	Animals and other organisms
☒	☐	Human research participants
☒	☐	Clinical data
☒	☐	Dual use research of concern

Methods

n/a	Involved in the study	
☒	☐	ChIP-seq
☒	☐	Flow cytometry
☒	☐	MRI-based neuroimaging

Eukaryotic cell lines

Policy information about cell lines

Cell line source(s)	hESCs (H1) were obtained from WiCell Research; mESCs were derived from a male, wild-type C57BL/6J mouse.
Authentication	The hESC line was not authenticated by us, but by the WiCell, which used Short Tandem Repeats (STRs) profiling to authenticate the cell lines (protocol available at https://www.wicell.org/home/characterization/identity/short-tandem-repeat-str/short-tandem-repeat-str.cmsx). The mESC line was authenticated by its competence for contributing to embryos when cultured on feeder cell-dependent condition followed by blastomere injection.

Mycoplasma contamination	All cell lines were tested negative during our routine qPCR-based mycoplasma tests.
Commonly misidentified lines (See ICLAC register)	No commonly misidentified cell lines were used in the study.

Animals and other organisms

Policy information about studies involving animals; ARRIVE guidelines recommended for reporting animal research

Laboratory animals	Tbxt-Δexon6/+ mouse model (Mus musculus, C57BL/6J) was generated through zygotic injection of CRISPR/Cas9 reagents. Other models were generated through blastocyst injection of engineered mESCs. Specially, C57BL/6J-albino female mice (Charles River laboratories, strain#493) were used for harvesting blastocysts. B6D2F1/J (Jackson laboratories, strain#100006) mice were used for harvesting blastocysts for fusion to tetraploid blastocysts. Wild-type C57BL/6J (strain#000664) mice were obtained from The Jackson laboratory. Mice were housed in the NYU Langone Health BSL1 barrier facility. 5-30 weeks old mice were used for breeding and phenotype analysis, with details on the age and sex described at the specific results section.
Wild animals	The study did not involve wild animals.
Field-collected samples	The study did not involve samples collected from field.
Ethics oversight	The study were performed following NYU Langone Health-approved ethical guidance and regulation on laboratory animals.

Note that full information on the approval of the study protocol must also be provided in the manuscript.
